# Performance Analyses and Improvements for the IEEE 802.15.4 CSMA/CA Scheme with Heterogeneous Buffered Conditions

**DOI:** 10.3390/s120405067

**Published:** 2012-04-19

**Authors:** Jianping Zhu, Zhengsu Tao, Chunfeng Lv

**Affiliations:** Department of Electronic, Information and Electrical Engineering, Shanghai Jiaotong University, No. 800, Minhang Road, Shanghai 200240, China; E-Mails: realwhitepig@sjtu.edu.cn (J.Z.); chunfenglv@sjtu.edu.cn (C.L.)

**Keywords:** performance evaluation, IEEE 802.15.4 CSMA/CA, heterogeneous unsaturated networks, OSTS/BSTS schemes, Markov chains, *M/G*/1/*K* theory, WSNs

## Abstract

Studies of the IEEE 802.15.4 Carrier Sense Multiple Access with Collision Avoidance (CSMA/CA) scheme have been received considerable attention recently, with most of these studies focusing on homogeneous or saturated traffic. Two novel transmission schemes—OSTS/BSTS (One Service a Time Scheme/Bulk Service a Time Scheme)—are proposed in this paper to improve the behaviors of time-critical buffered networks with heterogeneous unsaturated traffic. First, we propose a model which contains two modified semi-Markov chains and a macro-Markov chain combined with the theory of *M/G*/1/*K* queues to evaluate the characteristics of these two improved CSMA/CA schemes, in which traffic arrivals and accessing packets are bestowed with non-preemptive priority over each other, instead of prioritization. Then, throughput, packet delay and energy consumption of unsaturated, unacknowledged IEEE 802.15.4 beacon-enabled networks are predicted based on the overall point of view which takes the dependent interactions of different types of nodes into account. Moreover, performance comparisons of these two schemes with other non-priority schemes are also proposed. Analysis and simulation results show that delay and fairness of our schemes are superior to those of other schemes, while throughput and energy efficiency are superior to others in more heterogeneous situations. Comprehensive simulations demonstrate that the analysis results of these models match well with the simulation results.

## 1. Introduction

In recent years, wireless sensor networks (WSNs) have revolutionized the world of distributed systems and enabled many new applications. WSNs play more and more decisive roles in various aspects such as wide-range environmental surveillance, short-range health monitoring, inventory tracking, military locating *etc.*, and touch upon almost all aspects of our life, especially after the successful release of the IEEE 802.15.4 standard [1]. In addition to many of these diverse applications, WSNs have some burning questions. For example, all sensor networks are severely limited in terms of power consumption, which makes energy efficiency a very important design requirement. Besides energy requirements, other metrics of WSN systems such as service time, throughput and packet loss probability need to satisfy actual requirements of many real-time applications. Furthermore, quantities to be measured in the applications can be heterogeneous and unsaturated, such as detections of temperature and humidity in our periodic monitor application for a fire scene, which make existing homogeneous traffic analyses unrealistic. All types of transmitted information are exchanged between the ordinary nodes and the coordinator equally, which makes the access fairness among different types of nodes important. Therefore, understanding the characteristics of the buffered IEEE 802.15.4 networks with heterogeneous unsaturated traffic is essential in order to characterize the fundamental limitations of these networks and optimize deployed parameters accordingly.

In this work, we propose two novel access schemes named OSTS/BSTS to improve the heterogeneous performance of the time-critical network. First, we model these two slotted CSMA/CA schemes for a one-hop, beacon-enabled 802.15.4 star topology combining discrete time Markov chains and the theory of *M/G*/1/*K* queues. Nodes in this cluster contain finite buffers, followed heterogeneous and unsaturated traffic. Through these models, we can derive closed expressions for accessing probabilities, channel busy probabilities of clear channel assessments (CCAs), probability distribution of packet size, and then present the general performance metrics such as throughput, access delay and energy consumption. The distinguishing characteristic of these two schemes is that performance metrics are analyzed based on the overall point of view, which means transmitting processes take the dependent interactions of different types of nodes into account. To our best knowledge, there are few schemes dedicated to analyzing the buffered behaviors of networks with heterogeneous unsaturated traffic which have non-preemptive priority over each other, and this is the first comprehensive analysis and improvement for the IEEE 802.15.4 scheme in such a condition. Moreover, we propose comprehensive performance comparisons between our schemes and other schemes in which heterogeneous traffic is also bestowed non-priority, and find that the behaviors of our schemes are largely improved: delay and fairness of our models are superior to those of other schemes, while throughput and energy efficiency are superior to others in more heterogeneous situations.

The rest of this paper is structured as follows: Section 2 gives a summary of related works and analysis premise of our model. In Section 3, a brief overview of slotted CSMA/CA scheme of the IEEE 802.15.4 standard is described. OSTS/BSTS modeled by Markov chains and *M/G*/1/*K* queues in which nodes have finite buffers, following heterogeneous and unsaturated traffic are proposed in Section 4. In Section 5, an accurate analysis of throughput, delay and energy consumption is presented. Then, our model validations and comparisons of our model with other models using NS-2 simulator are provided in Section 6. Finally, concluding remarks and future work are presented in Section 7.

## 2. Related Works

Literature reviews presented here are three-fold: (1) references related to the performance analysis using Markov chain model; (2) references related to queuing performance analysis with buffered condition; (3) references related to performance analysis with heterogeneous traffic.

Among performance analyses of CSMA/CA backoff mechanisms using Markov chain models, a relatively early and comprehensive approach is presented in [2], which evaluates the performance of the IEEE 802.11 network. In [3], the 802.15.4 CSMA/CA adopting a similar Markov chain as [2] is analyzed, but using independent probability of sensing the channel instead of the independent probability of accessing the channel presented in [2]. A more intuitive and understandable Markov chain model is presented in [4], but the analysis results for the acknowledged and unacknowledged network deflects slightly with simulation results because of the adoption of a similar model as in [2]. Recently, new analyses taking retry limits into account are presented in [5] and [6], which adopting approximations to reduce complexity for the first time which do not match with simulations for using approximations instead of efforts to model exact behaviors. A hybrid channel access scheme using Markov chains presented in [7] combines CSMA/CA of IEEE 802.15.4 scheme with the Binary Exponential Backoff (BEB) scheme of IEEE 802.11. A certain decoupling approximation is adopted to identify an embedded Markov renewal process whose performance analysis yields a fixed point equation to derive saturation throughput in [8]. A mathematical discrete chain model is used to derive statistical distribution of the traffic in [9,10] to evaluate the access behavior of non-beacon-enabled and beacon-enabled CSMA/CA, respectively, which is based on a discrete chain but not a Markov chain, similar to [2]. As far as performance analysis is concerned, the only one which is based on bidirectional traffic of downlink and uplink is proposed in [11], adopting CSMA/CA Markov chain model building blocks. Two types of Markov chains are developed separately to describe the individual nodes and the channel state transition for determining the fractions of time that a node spends in different states which are then used to determine throughput and energy consumption characteristics in [12], and a geometric random distribution is used to present the number of backoff slots rather than the uniform random distribution as in [2]. Similar models as in [12] are proposed to evaluate the performance of multi-hop buffered IEEE 802.15.4 wireless networks in [13]. More accurate and comprehensive results are obtained for IEEE 802.15.4 transmission in [14] by introducing a new 4D Markov chain, which is used for determining the optimum value of the MAC attribute macSuperframeOrder (SO) required for saving energy, specifying an upper threshold on the number of nodes and the packet length required for achieving acceptable delay. All the aforementioned Markov models rely on solutions of various fixed point formulations without studying the existence and uniqueness of the fixed point, and only consider fixed length data packets without taking the variable packet lengths into account. A simple one-dimensional Markov chain model is proposed in [15] to solve these questions, which consider the existence and uniqueness of the fixed-point and the variable packet length for the saturated or unsaturated networks.

Queue-length distributions at arrival, departure and random epochs are proposed in detail in the serial schemes in [16–18], in which delay metrics are analyzed through various queue models in IEEE 802.11 networks. Delay analysis is also proposed in [19] with different contention window distribution to previous schemes, in which probability mass function (PMF) and probability generating function (PGF) are introduced to derive the performance of the buffered system. Queuing delay and achievable throughput of multi-hop networks are analyzed in [20]. Two Markov chain queuing models are developed to obtain solutions for packet delay and throughput distributions using IEEE 802.11 DCF (Distributed Coordination Function) in [21]. Delay character in non-preemptive priority queuing is presented by [22,23]. The scheme presented in [24] analyzes buffer characteristics of IEEE 802.15.4 queues for the first time, which uses discrete time Markov chains to present CSMA/CA scheme and the theory of *M/G*/1/*K* queues to character packet distributions.

Performance analyses of heterogeneous networks are mostly based on priorities, and the first performance analysis and modeling of 802.11 DCF with heterogeneous traffic based on fair contending chance presented in [25], which is based stochastic Markov chains, but these chains are not based on CSMA/CA scheme. The analytical model presented in [26] bestows a high priority to nodes whose contention windows are equal to one, which access the channel early than nodes whose contention windows are equal to two. A multi-level service differentiation scheme is introduced to analyze heterogeneous traffic in [27], which is not consistent with the fact that sensing measured variables of different nodes have the fair chance to be transmitted. Simple performance superposition of all nodes are used to model asymmetry character of IEEE 802.11 scheme in [28], which introduces post-backoff states to state transitions of Markov chain model to describe the unsaturated character. In [29], a 4-D discrete-time Markov chain model is proposed to derive the average service time and the service utilization factor of heterogeneous sensor networks, in which the devices can transmit data packets using CSMA/CA during the CAP or using the GTS during the CFP or both. Two types of nodes distributed over the area using two-dimensional homogeneous Poisson point processes in [30] are clustered two levels concluding different arrival rates, which introduces energy model to constrain arrival rates and minimize the overall cost based on a non-CSMA/CA scheme. The first CSMA/CA scheme analysis model of the IEEE 802.15.4 protocol for transmitting heterogeneous traffic of WSNs is presented in [31], which is based on the Markov chain model of [4], and the performance analysis is simply based on the superposition of two type nodes similar to that of [28]. A subtree-based iterative cascading scheduling mechanism and a workload-aware time slice allocation mechanism are proposed to improve the heterogeneous performance metrics such as energy consumption and latency in [32], and this W-MAC (Workload-Aware Medium Access Control) scheme can extend to dynamic networks, but control messages among parent nodes and children nodes consume a lot of energy. The so-called Differentiated Channel Access Scheme (DiffCA) is proposed in [33] to derive throughput fairness in heterogeneous networks by providing each node with an additional backoff counter, whose value varies according to the size of the packets. DiffCA achieves performance equilibrium resulting from packet size and accessing probability in terms of the service feasibilities, which is not the same fairness attribute as that of the scheme of [28] or our schemes. Two scheduling policies, which refer to a fixed priority scheduler and Earliest Deadline First (EDF) with late packet rescheduling, are implemented on top of a new CSMA/CA access protocol called Collect then Send burst Scheme (CoSenS) [34] to enhance the performance of throughput, end to end delay and reliability of heterogeneous WSN networks.

Comprehensive models adopting Markov chains and *M/G*/1/*K* queues are proposed to analyze and improve the performance of IEEE 802.15.4 CSMA/CA scheme. Simple and effective evaluations of throughput, delay and energy consumption are presented in a one-hop, star topology network which considers unsaturated and unacknowledged heterogeneous buffered uplink traffic, and adopting the beacon-enabled mode. Our system involves two different types of nodes, consisting of *N*_1_, *N*_2_ nodes to sense the variables of temperature and humidity in our periodic monitor application and transmit them to a sink, respectively. Packets arrive at the nodes for transmission according to a Poisson process with arrival rate of *λ*_1_ and *λ*_2_ for *N*_1_ and *N*_2_, respectively. System heterogeneity can be expressed as the node distributions, and the heterogeneity at the same node distribution can be denoted as the asymmetry which refers to the difference of packet arrival rates. Each node has a buffer with finite capacity *K* and each packet is fixed to *L* unit backoff period regardless of types. All nodes regardless of types are bestowed with equal opportunities to try to sense the channel without any priority or service differentiation, and traffic has non-preemptive priority over each other, which means that nodes or queues have fair chance to access the channel for a random period and current transmitting service cannot be interrupted by the new arrivals. We firstly propose two access schemes for our queuing transmission. The one is that a node which obtains the channel can transmit the queue header packet in its queue, and it can again contend for the channel with other nodes to transmit its remaining packets after completing this packet, denoted as one service a time scheme (OSTS). The other is that a node is allowed to transmit all packets with a burst mode once it successfully accesses the channel and reserves it, denoted as bulk service a time scheme (BSTS). Then, we analyze their behaviors and present the performance comparisons with other schemes. When the buffer is empty, the node will not attempt any transmission, while the buffer is full, the node will reject new packets coming from the upper layers.

The main contributions in this paper are threefold. Firstly, two novel schemes—OSTS/BSTS—are proposed to improve the behaviors of time-critical heterogeneous buffered networks with non-priority unsaturated traffic. Secondly, comprehensive models combining Markov chains and *M/G*/1/*K* queues are presented to analyze the heterogeneous performance of these schemes adopting a global viewpoint. Finally, performance comparisons are proposed to validate the superiority of these two schemes.

## 3. IEEE 802.15.4 Slotted CSMA/CA Mechanism

First, we briefly explain the slotted CSMA/CA mechanism of the IEEE 802.15.4 MAC [1]. In the beacon-enabled mode, a superframe is bounded by the transmission of a beacon frame and consists of an active part and an optional inactive part in which the coordinator may go to a low-power (sleep) mode. The active part consists of three parts: beacon, contention access period (CAP) and contention free period (CFP). Beacons, which commence at the beginning of the first slot, are used to synchronize attached nodes, identify Personal Area Networks (PANs) and describe the structure of the superframes. The CAP shall start immediately following the beacon and complete before CFP on a superframe slot boundary. All activities for nodes contending to access the channel are within this stage. The CFP, which slots are referred to as guaranteed time slots (GTS), is reserved by the PAN coordinator for dedicated access by some devices to ensure time-critical transmission, that is, the contention-free activities. The basic time unit of the MAC protocol is the duration of the so-called backoff period. Backoff slot boundaries of every node in the PAN are aligned with superframe slot boundaries of the PAN coordinator. The MAC sublayer shall ensure that the Physical (PHY) commences all of its transmissions on the boundary of a backoff period. That is, each time a node wishes to transmit data frames during the CAP, it must locate the boundary of the next slot period. Moreover, before accessing the channel, it should wait a random number of backoff slots. During this period, the node is in a sleeping state to save energy. After a random delay, two slot CCAs are carried out. In this work, we only take the CAP behavior of IEEE 802.15.4 superframe into account for performance analyses, and the CFP and its GTS are used to guarantee time-critical behaviors, such as on-time video streaming data flow in [35]. Of course, GTS scheme is also inferior in bandwidth utilization and the number of supported devices, which is improved largely in the enhanced Low Power Real Time (eLPRT) scheme [36].

**Figure 1.**
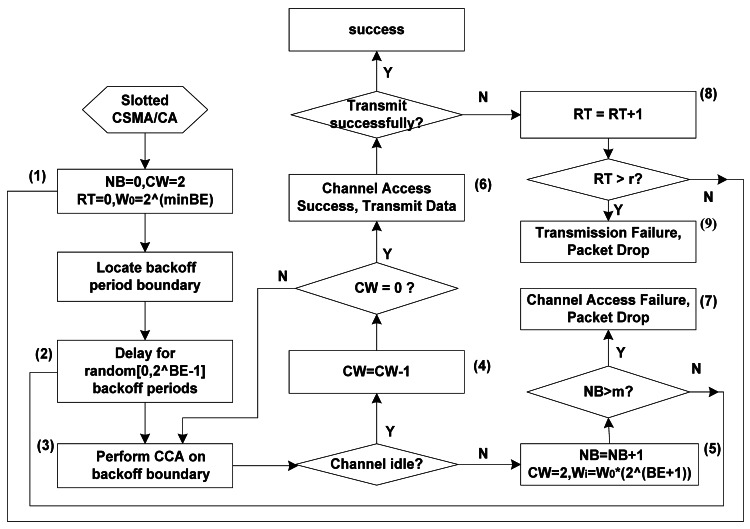
The slotted CSMA/CA mechanism of 802.15.4.

The scheme to be implemented before accessing the channel is illustrated in Figure 1 when a node has pending packets to transmit. In the slotted CSMA/CA of the IEEE 802.15.4, the MAC sublayer initializes four variables: number of backoff stage (*NB* = 0), contention window (*CW* = 2), retransmission stages (*RT* = 0) and backoff exponent (*BE* = *BE*_min_) (step 1). Then, the MAC sublayer delays for a random number of periods uniformly distributed in the first backoff range [0, 2*^BE^*^min^ − 1] (step 2). When the backoff counter is decreased to 0, the node performs the first CCA (step 3). If the channel is sensed idle after CCA1, *CW* decreases by one (step 4). If the channel is sensed idle after both consecutive CCAs, the node can access the channel successfully and then transmit packets (step 6). When the channel is sensed busy after either of the two CCAs, MAC sublayer will increase the value of *NB* and *BE* by one, respectively, and *CW* is reset to 2 (step 5). Backoff counters *W_i_* in which nodes randomly choose is increased exponentially accordingly (*W_i_ = W*_0_2^(^*^BE^*^+1)^). If *NB* is less than its max value *NB_m_*(*m*), the scheme must return to step 2, otherwise, the node will access the channel unsuccessfully and drop the packet (step 7). *NB* and *BE* values depend on the number of a packet's CCA failures. If the transmitting packet is in collision or transmitted unsuccessfully, the retransmission number of *RT* is increased by one (step 8). If *RT* exceeds its max value *RT_m_*(*r*), the packet is discarded due to transmission failure (step 9). Our system can monitor and detect objects periodically with enough nodes which transmit a lot of collected redundant information to coordinator without acknowledgement (ACK). The impacts of ACKs on the access behaviors can be ignored adopting the extra waiting time after a transmission, which is validated as shown in simulation results.

## 4. System Models

Before presenting system analytical models, several assumptions according to our actual applications are proposed.

1. ACK of MAC-level can be omitted for each packet for we consider two types of nodes transmitting packets to one sink (or coordinator) within one-hop star topology which is also presented in [12,13], and the coordinator can aggregate the received traffic from different types of nodes. The propagation signal effect can be disregarded for our distances among nodes are set to relatively close.
2. Empty probability denotes *μ*_0_ if there is no any packet in node buffer after a packet departure, which is not equal to the idle probability *P*_0_ at a random period. The node can go to sleep with a probability of *μ*_0_ if its buffer is empty at any one of such three situations: end of successful transmission; reaching maximum backoff stage; reaching retry limits.
3. Packet arrival process in buffers can be modeled as a Poisson process. Only header packets can contend for the channel every time, which leads to the channel contending analysis partly simple regardless of the queue distributions.
4. We modify that all nodes contending to the channel should decrease their backoff counters to initial values once one of them transmits successfully or packets are dropped due to channel access unsuccessfully or collision, avoiding nodes with low contention windows always capture the channel once they catch the channel in the case of competing for the channel simultaneously [37].

### 4.1. Markov Chains

In this section, two novel schemes with semi-Markov chain models describing slotted CSMA/CA scheme of IEEE 802.15.4 with retry limits and one macro-Markov chain model presenting macroscopic state transitions are proposed. The metrics of throughput, packet service time and energy consumption are partly determined by the network operating points *α, β* and *τ_n_* (*n* = 1, 2) which are derived through these models. We denote these two types of nodes as *N*_1_ and *N*_2_, respectively, for simplification.

**Figure 2.**
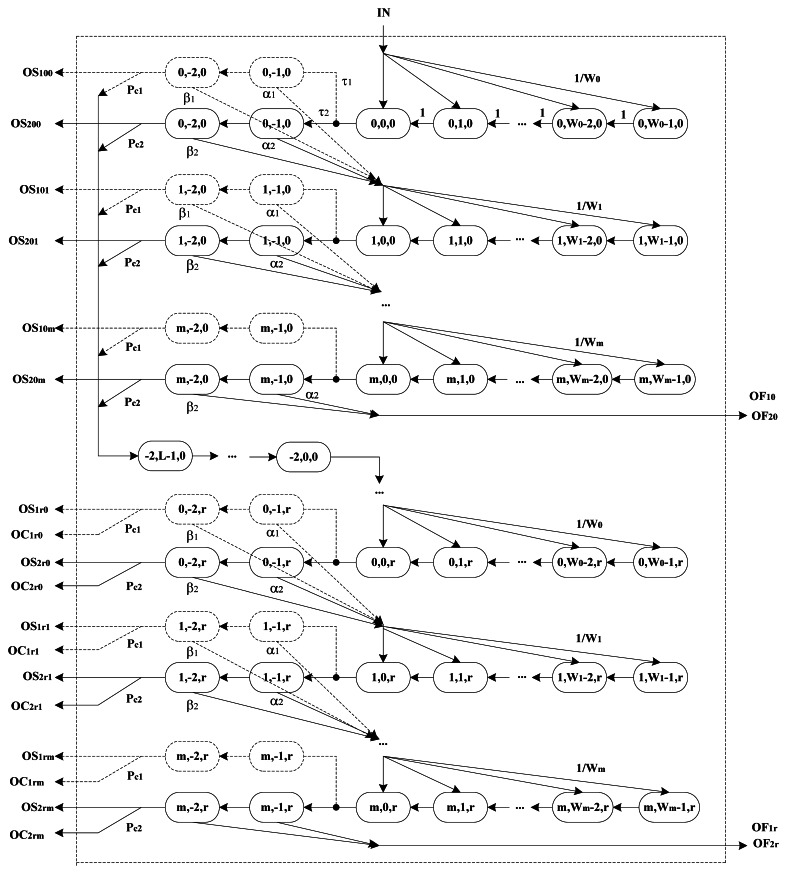
Markov chain model for slotted IEEE 802.15.4 CSMA/CA scheme. If a tagged node has packets to transmit at the next backoff slot with the probability *P_in_*, the node can access the channel with the slotted IEEE 802.15.4 CSMA/CA scheme. *P_in_* refers to the probability of *P*_1_ or *P*_2_. After random delay in range [0, *W*_0_ − 1], the node can perform CCA1 with a probability of *τ_n_* (*n* = 1, 2). It is denoted that probabilities *τ*_1,2_ located in the end of backoff is to demonstrate the paralleled access behavior of the different types of nodes since all nodes regardless of types must perform the backoff process.

First, we study the behaviors of one type of nodes using a three-dimensional Markov chain as in Figure 2. As interpreted above, we know CSMA/CA parameters are similar to each other for different type of nodes, so we can simplify different state transitions of these two types of nodes as one transition procedure of single type expect for the subscript. We define *s*(*t*)(*s*(*t*) ∈ (0, …, *m*)) as stochastic processes standing for backoff stage at time *t*, in which integer time *t* is corresponding to the beginning of slot times.

When the backoff stage increases to *m*, the node accesses the channel unsuccessfully and retries to access the channel if the buffer is not empty, otherwise it goes to a sleep state. We denote *s =* −1, *s =* −2 as the status of successful transmission and a failed one, respectively, which only appears at the derivation of normalized transition probability. Define *c*(*t*)(*c*(*t*) ∈ (−2, …, *W_i_* − 1)) as stochastic processes standing for backoff counter at time *t*. When the backoff counter decreases to 0, nodes sense the channel with probability *τ*_1_ for *N*_1_and *τ*_2_ for *N*_2_, respectively. Values *c* = −1, *c* = −2 stand for CCA1 and CCA2, respectively. Define *r*(*t*)(*r*(*t*) ∈ (0, …, *r*)) as states of retransmission counter at time *t*. Once transmitted unsuccessfully or when a collision occurs, pending packets can be retransmitted once more and *RT* is increased by one. When *RT* increases to *r*, the node retries to access the channel if its buffer is not empty, otherwise it goes into a sleep state [6]. After a successful transmission, the node also retries to access the channel if its buffer has another packet, otherwise it goes into a sleep state.

We denote actual state transitions by adopting solid ovals and solid arrows for the IEEE 802.15.4 CSMA/CA scheme using a Markov chain, such as *N*_2_ in Figure 2. In order to demonstrate the access procedure, we can show state transitions of the other node *N*_1_ using the same Markov scheme paralleled to the actual one with dashed ovals and dashed arrows which do not exist in the actual state transitions seen from Figure 2. All nodes, regardless of type, can sense the channel after random backoff periods with respective probability, and then access the channel with the probability of *τ*_1_ and *τ*_2_ after two successive idle backoff periods for *N*_1_ and *N*_2_, respectively. We denote *τ*_1_ and *τ*_2_ as presenting the parallel transition procedure for all nodes must perform the common backoff process. Output variables involved in Figure 2 can be expressed intuitively as follows: variables *OC*_1_*_r_*_0_ ∼ *OC*_1_*_rm_* and *OC*_2_*_r_*_0_ ∼ *OC*_2_*_rm_* are collision outputs at the maximal retry stage for *N*_1_ and *N*_2_, respectively. Variables *OF*_10_ ∼ *OF*_1_*_r_* and *OF*_20_ ∼ *OF*_2_*_r_* are accessing failure outputs for reaching limited number of backoff stage of *N*_1_ and *N*_2_, respectively. Variables *OS*_100_ ∼ *OS*_10_*_m_* to *OS*_1_*_r_*_0_ ∼ *OS*_1_*_rm_* and *OS*_200_ ∼ *OS*_20_*_m_* to *OS*_2_*_r_*_0_ ∼ *OS*_2_*_rm_* are the successful transmission outputs from the first retry stage to the maximal retry stage for *N*_1_ and *N*_2_, respectively. State transition probabilities for any one type of nodes associated with Markov chain of Figure 2 are:

(1) $$P(0,k,0)=P_{\text{in}}/W_{0},k=0\ldots W_{0}$$

(2) $$P(i,k,j|i,k+1,j)=1,i=0\ldots m,k=0\ldots W_{i},j=0\ldots r$$

(3) $$p(i,k,j|i-1,-2,j)=(\alpha_{n}+(1-\alpha_{n})\beta_{n})/W_{i},n=1,2$$

(4) $$P(0,k,j|i,-2,j-1)=(1-\alpha_{n})(1-\beta_{n})P_{\text{cn}}/W_{0},n=1,2$$

Equation (1) shows the connection between backoff procedure and macroscopic states. The backoff counter decreases one unit with probability one in every time interval, regardless of channel state shown as Equation (2). Equation (3) stands for the probability that a node goes to the next backoff stage after two failed CCA1 and CCA2 and selects a random counter in the next backoff stage. As long as *RT* is less than *r*, nodes choose to retransmit pending packets after any collision shown in Equation (4).

#### 4.1.1. Macroscopic State Transition for OSTS

**Figure 3.**
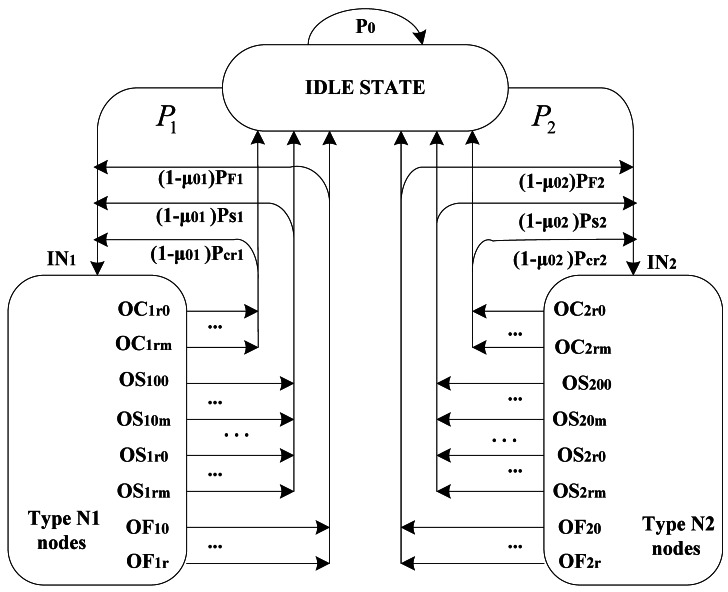
Macroscopic state transitions for OSTS scheme. Outputs within these blocks are those one-to-one corresponding outputs in Figure 2. A node goes to sleep with a probability of *μ*_0_*_n_* (*n* = 1, 2) after a transmission if its buffer is empty in the three situations: end of successful transmission, reaching maximum backoff stage or reaching retry limits, and it goes to another packet transmission with a probability of 1 − *μ*_0_*_n_* if it has other pending packets in these three situations. Channel keeps idle or sleeping with a probability of *P*_0_ if there is no any packet in any node. Nodes have packets to transmit at the next backoff slot with probabilities *P*_1_ and *P*_2_ for *N*_1_ and *N*_2_, respectively.

Packet queues in the node buffers are modeled as *M/G*/1/*K* queuing systems, and queues in either buffer have non-preemptive priority over each other. Packet arrivals follow a Poisson process with the average arrival rate of *λ*_1_ and *λ*_2_, respectively. The node which obtains the channel firstly can transmit the header packet in its queue, and it can again contend for the channel with other nodes to transmit its remaining packets after completing the current packet, denoted as one service a time scheme (OSTS). Macroscopic state transitions for OSTS are shown in Figure 3 with two types of nodes, in which output variables are intuitively the same as those of Figure 2.

Macroscopic states involving backoff procedures of both types of nodes follow the same algorithm as Figure 2, and we consider them as blocks. Node can go to sleep with the probability of *μ*_0_*_n_* (*n* = 1, 2) if there is no packet in the buffers after any one of such three situations: end of successful transmission; reaching maximum backoff stage; reaching retry limits. From Figures 2 and 3, we have the transition probabilities associated with Markov chains:

(5) $$P(\text{Idle}|m,0,j)=\mu_{0n}(\alpha_{n}+(1-\alpha_{n})\beta_{n})\;j\leq r$$

(6) $$P(\text{Idle}|i,0,r)=\mu_{0n}P_{\text{cn}}(1-\alpha_{n})(1-\beta_{n})\;i\leq m$$

(7) $$P(\text{Idle}|i,0,j)=\mu_{0n}(1-P_{\text{cn}})(1-\alpha_{n})(1-\beta_{n})$$

(8) $$P(\text{Idle}|\text{Idle})=P_{0}$$

(9) $$P(0,k,0|m,0,j)=(1-\mu_{0n})(\alpha_{n}+(1-\alpha_{n})\beta_{n})/W_{0}$$

(10) $$P(0,k,0|i,0,r)=(1-\mu_{0n})(1-\alpha_{n})(1-\beta_{n})P_{\text{cn}}/W_{0}$$

(11) $$P(0,k,0|i,0,j)=(1-\mu_{0n})(1-\alpha_{n})(1-\beta_{n})(1-P_{\text{cn}})/W_{0}$$

Equations (5)–(7) stand for the probability that a node goes to sleep after a departure if there is no packet in its buffer after unsuccessfully accessing the channel at each retry, unsuccessful transmission at the maximal retry or successful transmission, respectively. We denote the idle state as a transient state before the actual sleeping state. Equation (8) stands for probability that a node remains in the sleep state at a random slot. Equations (9)–(11) stand for the probability that a node goes to the next retransmission stage if there are other pending packets in the buffer after channel accessing failure, reaching retry limits and successful transmission, respectively. If a packet accesses the channel unsuccessfully or reaches its retry limit, this packet is discarded and the next packet in the buffer is transmitted.

Expressions of independent parameters *α, β* and *τ_n_* (*n* = 1, 2) can be derived from the formulas mentioned above. Denote *b_i,k,j_* = *P*{*s*(*t*), *c*(*t*), *r*(*t*) = *i, k, j*} as the steady-state probabilities of Markov chains, for *i* ∈ (−2, …, *m*), *k* ∈ (−2, …, *W_i_* − 1) and *j* ∈ (0, …, *r*). Owing to the Markov chain regularities and transition probability equations, we obtain:

(12) $$b_{i,k,j}^{n}=\frac{W_{i}-k}{W_{i}}b_{i,0,j}^{n}\;n=1,2$$

(13) $$b_{i,0,j}^{n}={\left(\alpha_{n}+(1-\alpha_{n})\beta_{n}\right)}^{i}b_{0,0,j}^{n}$$

(14) $$b_{0,0,j}^{n}=(1-\alpha_{n})(1-\beta_{n})P_{\text{cn}}\sum_{i=0}^{m}b_{i,0,j-1}^{n}={\left((1-\alpha_{n})(1-\beta_{n})P_{\text{cn}}\sum_{i=0}^{m}{\left(\alpha_{n}+(1-\alpha_{n})\beta_{n}\right)}^{i}\right)}^{j}b_{0,0,0}^{n}$$

Through normalized condition of Markov chains and steady-state probabilities according to each type of nodes, we obtain Equation (15). In this proposition, the distinguished character of this normalization probability is derived based on the view of overall instead of respective type of nodes which is related in [11,31]. Equation (16) contains the probability of backoff process, CCA1, CCA2, successful transmission process and unsuccessful transmission process, respectively:

(15) $$1=\sum b^{N_{1}}+\sum b^{N_{2}}+b_{\text{idle}}$$

(16) $$\sum b^{N_{n}}=\sum_{i=0}^{m}\sum_{k=0}^{W_{i}-1}\sum_{j=0}^{r}b_{i,j,k}^{n}+\sum_{i=0}^{m}\sum_{j=0}^{r}b_{i,-1,j}^{n}+\sum_{i=0}^{m}\sum_{j=0}^{r}b_{i,-2,j}^{n}+\sum_{j=0}^{r}\sum_{k=0}^{L-1}b_{-1,k,j}^{n}+\sum_{j=0}^{r}\sum_{k=0}^{L-1}b_{-2,k,j}^{n}$$

(17) $$\begin{array}{l} b_{\text{idle}}=P_{0}+\sum_{n=1}^{2}\mu_{0n}P_{\text{sn}}+\sum_{n=1}^{2}\mu_{0n}P_{\text{crn}}+\sum_{n=1}^{2}\mu_{0n}P_{\text{fn}}\\ =P_{0}+\sum_{n=1}^{2}\sum_{j=0}^{r}\sum_{i=0}^{m}\mu_{0n}(1-P_{\text{cn}})b_{i,-2,j}^{n}+\sum_{n=1}^{2}\sum_{i=0}^{m}\mu_{0n}P_{\text{cn}}b_{i,-2,r}^{n}+\sum_{n=1}^{2}\sum_{j=0}^{r}\mu_{0n}(\alpha_{n}+(1-\alpha_{n})\beta_{n})b_{m,0,j}^{n} \end{array}$$

Then, we can derive probability expressions of each block as follows. We assume there is no maximal delay exponent limitation for consideration of evaluation simplification:

(18) $$\sum b^{N_{1}}=b_{0,0,0}^{N_{1}}[\frac{1-{B_{1}}^{r+1}}{1-B_{1}}(\frac{1}{2}(\frac{1-{\left(2A_{1}\right)}^{m+1}}{1-2A_{1}}W_{0}+\frac{1-{A_{1}}^{m+1}}{1-A_{1}})+(2-\alpha_{1})\frac{1-{A_{1}}^{m+1}}{1-A_{1}}+L(1-\alpha_{1})(1-\beta_{1})(1-{A_{1}}^{m+1}))]$$

(19) $$\sum b^{N_{2}}=b_{0,0,0}^{N_{2}}[\frac{1-{B_{2}}^{r+1}}{1-B_{2}}(\frac{1}{2}(\frac{1-{\left(2A_{2}\right)}^{m+1}}{1-2A_{2}}W_{0}+\frac{1-{A_{2}}^{m+1}}{1-A_{2}})+(2-\alpha_{2})\frac{1-{A_{2}}^{m+1}}{1-A_{2}}+L(1-\alpha_{2})(1-\beta_{2})(1-{A_{2}}^{m+1}))]$$

$$A_{n}=\alpha_{n}+(1-\alpha_{n})\beta_{n},B_{n}=P_{\text{cn}}(1-\alpha_{n})(1-\beta_{n})\frac{1-{A_{n}}^{m+1}}{1-A_{n}}n=1,2$$

Equation (20) denotes the idle probability related with queue character, in which *P*_0_ is the probability that a node remains in a sleeping state without any packet arrival in a random slot time. From Equation (20) and Figure 3, idle probability consists of four parts which refers to the probability of no packet presenting in any node, successful transmission probability of either node, unsuccessful transmission probability for retry limits of either node and unsuccessful access probability for backoff stage limits of either node respectively, which is also related in [6]:

(20) $$b_{\text{idle}}=P_{0}+(\frac{{A_{1}}^{m+1}(1-{B_{1}}^{r+1})}{1-B_{1}}+P_{c1}(1-{A_{1}}^{m+1}){B_{1}}^{r}+(1-P_{c1})\frac{\left(1-{A_{1}}^{m+1}(1-{B_{1}}^{r+1}\right)}{1-B_{1}})\mu_{01}b_{0,0,0}^{N_{1}}+(\frac{{A_{2}}^{m+1}(1-{B_{2}}^{r+1})}{1-B_{2}}+P_{c2}(1-{A_{2}}^{m+1}){B_{2}}^{r}+(1-P_{c2})\frac{\left(1-{A_{2}}^{m+1})(1-{B_{2}}^{r+1}\right)}{1-B_{2}})\mu_{02}b_{0,0,0}^{N_{2}}$$

Substituting Equations (18)–(20) into Equation (15), we can obtain that the normalized probability is related to two Markov chain variables $b_{0,0,0}^{N_{1}}$ and $b_{0,0,0}^{N_{12}}$, along with three queue variables *μ*_01_, *μ*_02_ and *P*_0_. Variables $b_{0,0,0}^{N_{1}}$ and $b_{0,0,0}^{N_{12}}$ related with Markov chains can be derived through previous analysis. Nodes have packets to transmit at the next backoff slot with probabilities *P*_1_ and *P*_2_ for *N*_1_ and *N*_2_ respectively, which means that nodes in *N*_1_ can start to access the channel at the boundary of the next slot with probability *P*_1_ if there are no new packet arrivals of other type of nodes. The next transmission probability *P*_2_ for *N*_2_ is derived as the similar way. According to the Markov blocks of the macroscopic state transition in Figure 3, we can derive relations between semi-Markov models of single CSMA/CA scheme and macro-Markov model of integral channel states, that is, the probability *P_in_* in Figure 2 and Equation (1) can be presented as *P*_1_ or *P*_2_ in Figure 3 intuitively. Consequently, we can express all components of Equation (15) as functions of variable *Q*_*L*_0__ which refers to idle state length in the state transition.

In such a way, we can derive all parameters in the system using a numerical method that solves the non-linear system equations given by Equations (15), (21), (22), in which *T_trn_*(*Z*) and $\bar{{\text{T}}_{\text{trn}}}$ (*n* = 1, 2) are denoted as the distribution and mean value of packet access time, respectively:

(21) $$\begin{array}{l} b_{0,0,0}^{N_{1}}=Q_{L_{0}}P_{1}+(1-\mu_{01})P_{s1}+(1-\mu_{01})P_{cr1}+(1-\mu_{01})P_{f1}\\ =Q_{L_{0}}\frac{\lambda_{1}\bar{T_{tr1}}}{\mu_{01}+\lambda_{1}\bar{T_{tr1}}}(1-\frac{\lambda_{2}\bar{T_{tr2}}}{\mu_{02}+\lambda_{2}\bar{T_{tr2}}})+(1-\mu_{01})(\sum_{j=0}^{r}\sum_{i=0}^{m}(1-P_{c1})b_{i,-2,j}^{N_{1}}+\sum_{i=0}^{m}P_{c1}b_{i,-2,j}^{N_{1}}+\sum_{j=0}^{r}A_{1}b_{m,0,j}^{N_{1}}) \end{array}$$

(22) $$\begin{array}{l} b_{0,0,0}^{N_{2}}=Q_{L_{0}}P_{2}+(1-\mu_{02})P_{s2}+(1-\mu_{02})P_{cr2}+(1-\mu_{02})P_{f2}\\ =Q_{L_{0}}\frac{\lambda_{2}\bar{T_{tr2}}}{\mu_{02}+\lambda_{2}\bar{T_{tr2}}}(1-\frac{\lambda_{1}\bar{T_{tr1}}}{\mu_{01}+\lambda_{1}\bar{T_{tr1}}})+(1-\mu_{02})(\sum_{j=0}^{r}\sum_{i=0}^{m}(1-P_{c2})b_{i,-2,j}^{N_{2}}+\sum_{i=0}^{m}P_{c2}b_{i,-2,r}^{N_{2}}+\sum_{j=0}^{r}A_{2}b_{m,0,j}^{N_{2}}) \end{array}$$

We present probability expressions of *P*_1_ and *P*_2_ here for an early time, which are deduced elaborately in Section 4.2. Probability *P*_0_ that there is no packet to send in a random slot, and probabilities *μ*_01_, *μ*_02_ which means that the queue become empty after a departure of *N*_1_ and *N*_2_ respectively can be derived through the queuing theory analyzed in the next section.

#### 4.1.2. Macroscopic State Transition for BSTS

The second scheme BSTS, denoting s bulk service a time scheme, means that a node is allowed to transmit all packets in its buffer with a burst mode once it successfully obtains the channel and reserves it. In this scheme, transmission packet length is simply considered as *KL* units of backoff period. Packets in the buffer queues are transmitted entirely once the node acquires the channel, and then it goes to idle state directly, which means that the node goes to idle (or sleep) state with the probability of 1 after any one of such three situations: end of successful transmission, reaching maximum backoff stage and reaching retry limits.

**Figure 4.**
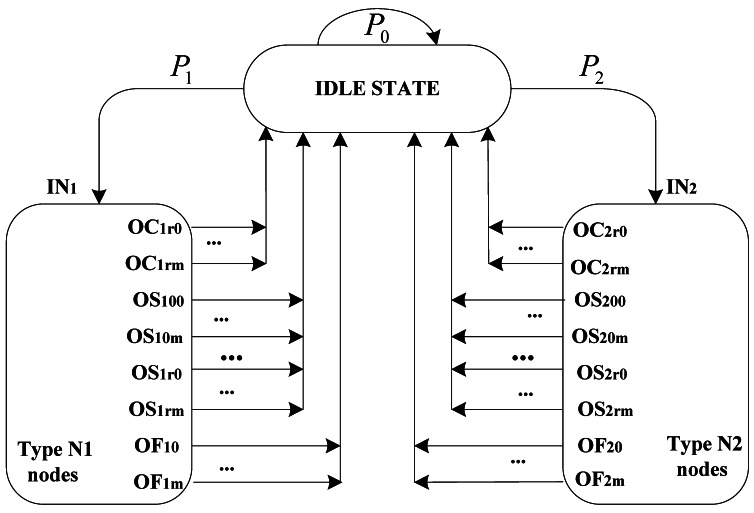
Macroscopic state transitions for BSTS scheme. Outputs within these blocks are those one-to-one corresponding outputs in Figure 2. A node goes to sleep with a probability of one after all packets transmitted with a burst way at three situations: end of successful transmission, reaching maximum backoff stage or reaching retry limits. Channel keeps idle state with probability of *P*_0_ if there is no any packet in any node. Nodes have packets to transmit at the next backoff slot with probabilities *P*_1_ and *P*_2_ for *N*_1_ and *N*_2_, respectively.

Performance analysis of this scheme is similar to that of OSTS except for setting the parameter *μ*_0_*_n_* to one. We can modify the scheme of OSTS for taking no account of parameter *μ*_0_*_n_*, which simplifies the normalized steady-state probability closed expressions. According to Equations (5)–(7), the transition probability expressions associated macroscopic Markov chain of Figure 4 can be derived by setting parameter *μ*_0_*_n_* to one, and then Equations (9)–(11) can be omitted accordingly. Other expression definitions correlated with BSTS are similar to OSTS, such as Equations (15)–(20), and thus, the state transition behaviors of this CSMA/CA scheme can be considered as one packet in buffer. This packet is not a real IEEE 802.15.4 packet, but a considered packet sequence which is been successively transmitted in the buffer. Its performance can be easily derived. Equations (21) and (22) can be simplified as follows:

(23) $$b_{0,0,0}^{N_{1}}=Q_{L_{0}}P_{1}=Q_{L_{0}}(1-\lambda_{2}/KL)\lambda_{1}/KL$$

(24) $$b_{0,0,0}^{N_{2}}=Q_{L_{0}}P_{2}=Q_{L_{0}}(1-\lambda_{1}/KL)\lambda_{2}/KL$$

### 4.2. Queuing Models

We denote *T_trn_*(*Z*) and $\bar{{\text{T}}_{\text{trn}}}$ (*n* = 1, 2) as the distribution and mean value of packet access time, respectively. Each queue can accommodate *K* packets, and those arrivals that find *K* present in the queue will drop. Packet length distribution of each node can be derived independently and respectively because packets of two types arrive at respective queues of two types independently. We can denote *p_jk_* as the state transition probability that the queue length changes from *j* to *k* immediately after a packet departure. Probability *p_jk_* is independent of *K* and *n*, and *p*_0_*_k_* = *a_k_* (0 ≤ *k* ≤ *K* − 2), *p_jk_* = *a_k_*_-_*_j_*_+1_ (0 ≤ *j* ≤ *K* − 1) in which *a_kn_*(*n* = 1, 2) is the probability of *k* packet arrivals to the two queues during the packet access time respectively, which is presented as follows [21,24,38,39]:

(25) $$\begin{array}{l} a_{\text{kn}}=\int_{0}^{\infty }\frac{{\left(\lambda_{n}t\right)}^{k}}{k!}e^{-\lambda_{n}t}dT_{\text{trn}}(t)\;k=0,1,2,\ldots n=1,2\\ A_{\text{kn}}(Z)=\sum_{k=0}^{\infty }a_{\text{kn}}Z^{k}=\int_{0}^{\infty }e^{-t\lambda_{n}(1-Z)}dT_{\text{trn}}(t)=T_{\text{trn}}^{*}(\lambda_{n}-Z\lambda_{n}) \end{array}$$

We also denote *A_kn_*(*Z*) as the PGF for the number of packet arrivals at the queues during the packet service time, as shown in Equation (25). We denote *μ_kn_* as the steady-state probability that there are *k* packets in queue immediately after a packet departure [21,24,38,39]:

$$\mu_{\text{kn}}=\sum_{j=0}^{K-1}\mu_{\text{jn}}\;p_{\text{jk}}\;0\leq k\leq K-1;\sum_{k=0}^{K-1}\mu_{\text{kn}}=1;$$

And then, steady-state equations for state transitions are given as follows [21,24]:

(26) $$\mu_{\text{kn}}=\mu_{0n}a_{\text{kn}}+\sum_{j=1}^{k+1}\mu_{\text{jn}}a_{k-j+1}^{n}\;0\leq k\leq K-2$$

(27) $$\mu_{K-1}^{n}=\mu_{0n}\sum_{k=K-1}^{\infty }a_{\text{kn}}+\sum_{j=1}^{K-1}\mu_{\text{jn}}\sum_{k=K-j}^{\infty }a_{\text{kn}}$$

We find Equation (27) is redundant, and Equation (26) provides *K* independent equations for *K* unknowns *μ_kn_* (0 ≤ *k* ≤ *K* − 1), so we can solve the system using an efficient algorithm as introducing the substitution $\mu_{\text{kn}}^{\prime }=\mu_{\text{kn}}/\mu_{0n}(0\leq k\leq K-1)$, which is easy to see from Equation (24) that $\mu_{\text{kn}}^{\prime }$ (0 ≤ *k* ≤ *K* − 1) can be recursively calculated as follows:

(28) $$\mu_{0n}^{\prime }=1;\mu_{(k+1)n}^{\prime }=\frac{1}{a_{0n}}(\mu_{\text{kn}}^{\prime }-\sum_{j=1}^{k}\mu_{\text{jn}}^{\prime }a_{k-j+1}^{n}-a_{\text{kn}})\;0\leq k\leq K-2$$

From above queue expressions, we can derive the probability *μ*_0_*_n_* that the queue is empty immediately after a departure, which means any one of such three situations: end of successful transmission; reaching retries limits; reaching maximum backoff stage [21,24]:

(29) $$\mu_{0n}=\frac{1}{\sum_{k=0}^{K-1}\mu_{\text{kn}}^{\prime }}$$

The probability *P*_0_ that the queue is empty at arbitrary time can be derived unlike the way of the probability *μ*_0_*_n_*, but these two probabilities must both comply with the steady-state equations. Packet can be accepted by the queue with the probability of (1 − *P_Kn_*), in which $P_{\text{Kn}}=1-\sum_{k=0}^{K-1}\mu_{\text{kn}}$, and then *P*_0_ can be derived as follows:

(30) $$P_{0}=1-\frac{\lambda_{1}\bar{T_{tr1}}}{\mu_{01}+\lambda_{1}\bar{T_{tr1}}}(1-\frac{\lambda_{2}\bar{T_{tr2}}}{\mu_{02}+\lambda_{2}\bar{T_{tr2}}})-\frac{\lambda_{2}\bar{T_{tr2}}}{\mu_{02}+\lambda_{2}\bar{T_{tr2}}}(1-\frac{\lambda_{1}\bar{T_{tr1}}}{\mu_{01}+\lambda_{1}\bar{T_{tr1}}})$$

We can derive the closed expressions for system depiction by substituting Equations (29), (30) to Equations (20)–(22) and (15) can be solved by these expressions adopting a mathematic method.

## 5. Performance Analysis

System operating points are determined by parameters *α, β* and *τ_n_* (*n* = 1, 2), which can be derived from the expressions related above. Actually, operating points used for channel state depictions are related with those packets contending to acquire the channel, which are the exact header packets of these queues in one contending period cycle (CPC). Contending processes have been independent of other remaining packets in the queue buffers. The probability that a node attempts to sense the channel for CCA1 in a randomly chosen time slot is denoted by *τ*, representing backoff counter decreased to 0:

(31) $$\tau_{n}=\sum_{i=0}^{m}\sum_{j=0}^{r}b_{i,0,j}^{n}=\frac{1-{A_{n}}^{m+1}}{1-A_{n}}\frac{1-{B_{n}}^{r+1}}{1-B_{n}}b_{0,0,0}^{n}\;n=1,2$$

We analyze the medium behavior based on every CPC for simplification. When the channel is sensed busy after CCA1 with probability *α*_1_ for *N*_1_ due to data transmission of other nodes, it means that at least one of (*N*_1_ − 1) remaining nodes transmits in the same slot with the current transmitting node and none of *N*_2_ transmit, or none of remaining *N*_1_ − 1 transmits and at least one of *N*_2_ transmits [6,12]. Probability *α*_2_ for *N*_2_ can be derived in a similar way. In this way, channel sensing probabilities are independent of types such as Equation (32). Probabilities *β*_1_ and *β*_2_, which refer to the channel sensed busy after CCA2 for *N*_1_ and *N*_2_ respectively, can be derived in the same way. It can be simplified to Equation (33) which means at least one of *N*_1_ and *N*_2_ transmits in current slot:

$$\begin{array}{l} \alpha_{1}=(L+t_{\text{ex}})\{(1-{\left(1-\tau_{1}\right)}^{N_{1}-1})(1-\alpha_{1})(1-\beta_{1}){\left(1-\tau_{2}\right)}^{N_{2}}+{\left(1-\tau_{1}\right)}^{N_{1}-1}\left(1-{\left(1-\tau_{2}\right)}^{N_{2}})(1-\alpha_{2})(1-\beta_{2})\right\}\\ \alpha_{2}=(L+t_{\text{ex}})\{(1-{\left(1-\tau_{1}\right)}^{N_{1}})(1-\alpha_{1})(1-\beta_{1}){\left(1-\tau_{2}\right)}^{N_{2}}+{\left(1-\tau_{1}\right)}^{N_{1}}(1-{\left(1-\tau_{2}\right)}^{N_{2}-1})(1-\alpha_{2})(1-\beta_{2})\} \end{array}$$

(32) $$\alpha =\frac{\tau_{1}\alpha_{1}+\tau_{2}\alpha_{2}}{\tau_{1}+\tau_{2}}$$

$$\beta_{1}=\beta_{2}=1-{\left(1-\tau_{1}\right)}^{N_{1}}{\left(1-\tau_{2}\right)}^{N_{2}}$$

(33) $$\beta =\frac{\tau_{1}\beta_{1}+\tau_{2}\beta_{2}}{\tau_{1}+\tau_{2}}$$

**Figure 5.**
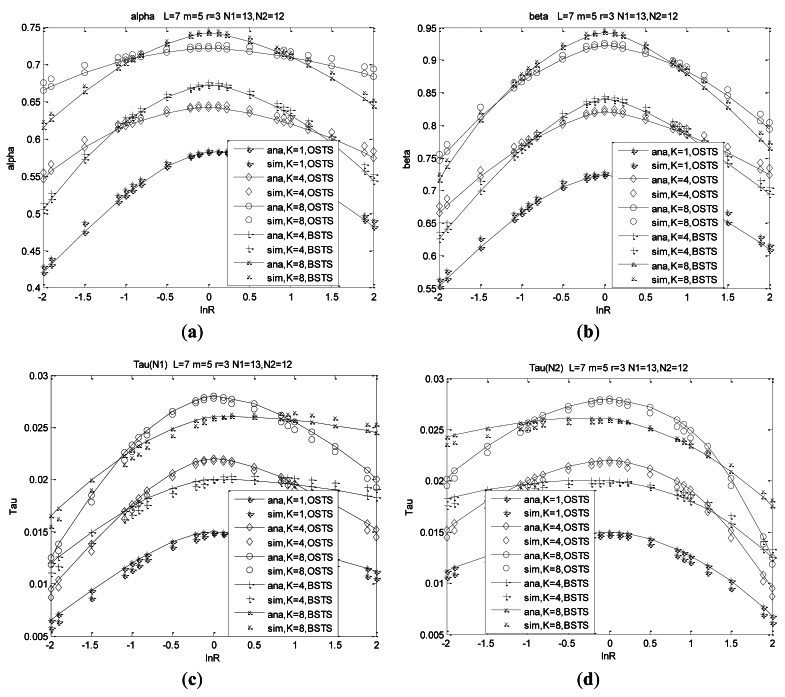
The behavior of parameters *α, β* and *τ_n_* (*n* = 1, 2) in heterogeneous system. (**a**) Relations of *α* with ln*R*; (**b**) Relations of *β* with ln*R*; (**c**) Relations of *τ*_1_ with ln*R*; (**d**) Relations of *τ*_2_ with ln*R*.

Network operating points determined by carrier sensing probability *τ_n_* (*n* = 1, 2) and busy channel probabilities *α, β* are derived from three non-linear expressions of Equations (31)–(33) using a numerical fixed point method. Figure 5(a–d) illustrate the characteristics of parameters *α, β* and *τ_n_* (*n* = 1, 2) as functions of R = *λ*_1_/*λ*_2_, in which simulation setup and simulation parameters are presented later in Section 6.

Since parameters *α, β* and *τ_n_* are only related to the exact contending packets in the medium, their characters are similar to those of nodes without buffers in our previous analysis. We only analyze operating points for a network size of 25 nodes with the most heterogeneous traffic, and the other metrics such as throughput, service delay or energy consumption are also taken the least asymmetric traffic condition into account in later analyses.

In the most heterogeneous condition which refers to the number of two type nodes are comparable to each other, we observe that channel accessing character is dominated by the difference of the two arrival rates. Channel is sensed busy with smaller probability for there are small total pending packets when traffic rate *λ*_1_ is much smaller than *λ*_2_, such as ln*R* = −2 and *vice versa*. Probability *α* of channel being sensed busy for CCA1 increases as the system dissimilarity or asymmetry decreases. Asymmetry refers to the difference of arrival rates *λ*_1_ and *λ*_2_. Probability *α* arrives at its peak value when traffic rate *λ*_1_ is equal to *λ*_2_ at different node distribution, and *α* increases with the queue length, meaning the buffer capacity. Channel accessing behavior of BSTS is the same as that of OSTS when *K* = 1. For BSTS scheme, we adopt the fragment indication message passing (FIMP) algorithm presented in [40], in which we can only retransmit the indicated failed packets instead of the whole packets in the queue to reduce energy or delay consumption. We observe that *α* in BSTS increases more rapidly than that of OSTS with the difference of arrival rates decreasing, that is, the asymmetry in the same node distribution. Due to pending packets increased with the asymmetry decreasing, more time is consumed to transmit or retransmit such long packets accumulated at the boundary of super-frame for BSTS, consequently, the transmitting efficiency of BSTS decreases. Moreover, *α* for BSTS becomes higher than that for OSTS with the asymmetry decreasing for more time is required to wait for transmit the failed indicated fragments which increase largely with asymmetry decreasing.

Probabilities *β* and *τ_n_* (*n* = 1, 2) are analyzed in the same way as *α*. Probability *β*, for a channel sensed busy for the second CCA, increases to a high value with the difference of arrival rates decreasing, and reaches its peak in the case of *λ*_1_ = *λ*_2_. Decreasing the difference of arrival rates, *β* for BSTS is higher than that for OSTS. One reason is that the difference of arrival rates increases, meaning that pending packets are almost dominated by the higher rate nodes. Less time is consumed to transmit homogeneous packets regardless of its length, which leads to BSTS suitability. Moreover, failed packets or retransmission packets of BSTS will increase rapidly with asymmetry decreasing due to nodes consume much time to wait for detecting failed indicated packets for adopting FIMP scheme. Consequently, transmission probabilities *τ*_1_ and *τ*_2_ of BSTS are less slightly than those of OSTS under more asymmetry conditions.

From these figures, we observe that accessing behaviors are determined by the node distribution and system asymmetry. Analysis results are consistent with simulation results at more symmetry conditions for a great extent, while those of more asymmetry cases are inconsistent with analysis results slightly shown in Figure 5(a–d), and these deflections can be susceptive in our system design.

### 5.1. Throughput Analysis

We denote *S* as normalized throughput, which is defined as the fraction of time the channel is used to successfully transmit payload bits in every CPC. A random chosen slot consists of three possibilities: fraction of time for successful transmission, fraction of time for collision and fraction of time for idle or sleeping. We calculate each time fraction for deriving *S*:

(34) $$\begin{array}{c} P_{tr1}=(1-{\left(1-\tau_{1}\right)}^{N_{1}})(1-\alpha_{1})(1-\beta_{1}){\left(1-\tau_{2}\right)}^{N_{2}}\\ P_{tr2}=(1-{\left(1-\tau_{2}\right)}^{N_{2}})(1-\alpha_{2})(1-\beta_{2}){\left(1-\tau_{1}\right)}^{N_{1}} \end{array}$$

Successful transmission probability *P_s_* is given by the probability that exactly one node transmits on the channel, conditioned on the fact that at least one node transmits:

(35) $$\begin{array}{c} P_{s1}=\frac{N_{1}\tau_{1}{\left(1-\tau_{1}\right)}^{N_{1}-1}(1-\alpha_{1})(1-\beta_{1}){\left(1-\tau_{2}\right)}^{N_{2}}}{P_{tr1}}=\frac{N_{1}\tau_{1}{\left(1-\tau_{1}\right)}^{N_{1}-1}}{1-{\left(1-\tau_{1}\right)}^{N_{1}}}\\ P_{s2}=\frac{N_{2}\tau_{2}{\left(1-\tau_{2}\right)}^{N_{2}-1}(1-\alpha_{2})(1-\beta_{2}){\left(1-\tau_{1}\right)}^{N_{1}}}{P_{tr2}}=\frac{N_{2}\tau_{2}{\left(1-\tau_{2}\right)}^{N_{2}-1}}{1-{\left(1-\tau_{2}\right)}^{N_{2}}} \end{array}$$

Probabilities that nodes encounter the collisions in a random slot are not similar to successful transmission probabilities as follows:

(36) $$\begin{array}{c} P_{c1}={\left(1-\tau_{2}\right)}^{N_{2}}\sum_{k=2}^{N_{1}}\left(\begin{array}{l} N_{1}\\ k \end{array}\right){P_{tr1}}^{k}{\left(1-P_{tr1}\right)}^{N_{1}-k}+\sum_{m=1}^{N_{1}}\left(\begin{array}{l} N_{1}-1\\ m \end{array}\right){P_{tr1}}^{m}{\left(1-P_{tr1}\right)}^{N_{1}-1-m}+\sum_{l=1}^{N_{2}}\left(\begin{array}{l} N_{2}\\ l \end{array}\right){P_{tr2}}^{l}{\left(1-P_{tr2}\right)}^{N_{2}-l}\\ P_{c2}={\left(1-\tau_{1}\right)}^{N_{1}}\sum_{k=2}^{N_{2}}\left(\begin{array}{l} N_{2}\\ k \end{array}\right){P_{tr2}}^{k}{\left(1-P_{tr2}\right)}^{N_{2}-k}+\sum_{m=1}^{N_{1}}\left(\begin{array}{l} N_{1}\\ m \end{array}\right){P_{tr1}}^{m}{\left(1-P_{tr1}\right)}^{N_{1}-m}+\sum_{l=1}^{N_{2}}\left(\begin{array}{l} N_{2}-1\\ l \end{array}\right){P_{tr2}}^{l}{\left(1-P_{tr2}\right)}^{N_{2}-1-l} \end{array}$$

Thus, throughput expression *S* is derived through these three parts:

(37) $$S=\frac{P_{tr1}P_{s1}E_{1}[P]+P_{tr2}P_{s2}E_{2}[P]}{(1-P_{tr1}P_{s1}-P_{tr2}P_{s2}-P_{c1}-P_{c2})\sigma +(P_{tr1}P_{s1}+P_{tr2}P_{s2})T_{s}+(P_{c1}+P_{c2})T_{c}}$$

*E_n_*[*P*] is the average packet payload size in number of slots, and average payload information content successfully transmitted in a slot time is *P_trn_ P_sn_ E_n_*[*P*]. At the same time, we can derive three parts in a random slot using the separate probabilities. Successful transmission probability is *P_tr_*_1_*P_s_*_1_ + *P_tr_*_2_*P_s_*_2_ and collision transmission probability is *P_c_*_1_ + *P_c_*_2_, respectively. *T_s_* is average time that the channel is sensed busy due to a successful transmission, while *T_c_* is time that the channel is sensed busy due to collision. If there is at least one transmission in a random slot, remaining time in a slot is idle period with a length of (1 − *P_tr_*_1_*P_s_*_1_ − *P_tr_*_2_*P_s_*_2_ − *P_c_*_1_ − *P_c_*_2_)*σ*. According to a time-critical and energy efficient redundancy network, the coordinator does not need to acknowledge each packet. Since there is no collision detection in the CSMA/CA mechanism, the channel remains awake after successful and failed state for several slot durations. Thus, after each transmission, we assume that a node keeps at least two slots receiving before next transmission in this occasion. *E*[*P*], *T_s_, T_c_*, and *σ* are independent of system parameters, but, *P_tr_*_1,2_, *P_s_*_1,2_ and *P_c_*_1,2_ depend on operating point parameters *α, β* and *τ*_1,2_ as shown in Equations (31)–(33). Expressions of *T_s_* and *T_c_* are:

(38) $$T_{s}=2\left\lceil t_{\text{CCA}}\right\rceil +\left\lceil t_{s}\right\rceil +\left\lfloor t_{\text{ex}}\right\rfloor ,T_{c}=2\left\lceil t_{\text{CCA}}\right\rceil +\left\lceil t_{c}\right\rceil +\left\lfloor t_{\text{ex}}\right\rfloor$$

Assume *t_s_* = *t_c_* = *t_L_*, where *t_CCA_, t_s_, t_c_* and *t_ex_* are durations for performing a CCA, transmitting a *L*-slot packet successfully, transmitting a *L*-slot packet unsuccessfully and keeping waiting for extra slots, respectively. Without ACK and waiting for ACK, the successful transmission length is as same as the failure one, and delay, energy consumption and throughput design procedures are relatively simple compared to those of different transmission length. After a successful or failed transmission, extra waiting time during which the channel becomes clear again is determined by the practical situation.

### 5.2. Delay Analysis

In low-rate wireless applications, packet service delay is also an important metric, and we pay more attention to improving the performance of delay in our time-critical applications. Generally, total delay in a communication network includes processing delay, queuing delay, access delay, and propagation delay. In this paper, we focus on average packet service delay which consists of the delay in queue waiting and delay for accessing the channel. Access delay is the time from the instant which the packet is at the head of its MAC queue and ready to be transmitted to the instant when coordinator receives packet, which is also elaborately discussed by many papers such as [6,41,42]. We can denote the PGF of access delay as *T_tr_*(*Z*). The time for packet waiting in its queue is derived through the queuing theory such as [11,21,24]. The PGF of queuing delay can be denoted as *T_q_*(*Z*). We analyze the accessing character of the buffered system and derive the delay metric accordingly. Empty probabilities *μ*_0_ immediately after a departure and *P*_0_ at a random period are of our consideration besides the queue distribution involving the tagged packet.

#### 5.2.1. Access Delay *T_tr_*(*Z*)

The PGF of access delay consists of three factors as shown in Equation (39): the first part is the PGF of successful transmission, the second part is the PGF of access failure and the last is the PGF of failure transmission for reaching retry limits. Transmission commences as the channel being sensed idle for two CCAs, as factor *Z*^2^ in each part of Equation (39). A packet is transmitted successfully with probability of (1 − *α*)(1 − *β*)(1 − *P_c_*) and transmitted unsuccessfully with probability of (1 − *α*)(1 − *β*)*P_c_* after *j* collisions, respectively. Each backoff stage follows by a short turnaround period for we consider nodes sleeping in backoff decrement process, and we denote it as *T_ta_* shown in Equation (39):

(39) $$\begin{array}{l} T_{\text{trn}}(Z)=Z^{L+T_{\text{ex}}}(1-\alpha_{n})(1-\beta_{n})(1-P_{\text{cn}})\sum_{j=0}^{r}{\left(\sum_{i=0}^{m}{\left((1-\alpha_{n})(1-\beta_{n})\right)}^{i}P_{c}Z^{L+T_{\text{ex}}}\right)}^{j}{\left(Z^{2+T_{\text{ta}}}\sum_{i=0}^{m}B_{i}(Z)F_{i}(Z)\right)}^{j+1}\\ +\sum_{j=0}^{r}{\left(\sum_{i=0}^{m}{\left((1-\alpha_{n})(1-\beta_{n})\right)}^{i}P_{c}Z^{L+T_{\text{ex}}}\right)}^{j}A^{m+1}{\left(Z^{2+T_{\text{ta}}}\sum_{i=0}^{m}B_{i}(Z)F_{i}(Z)\right)}^{j}B_{m+1}(Z)F_{m+1}(Z)\\ +{\left(\sum_{i=0}^{m}{\left((1-\alpha_{n})(1-\beta_{n})\right)}^{i}P_{c}Z^{L+T_{\text{ex}}}\right)}^{r+1}{\left(Z^{2+T_{\text{ta}}}\sum_{i=0}^{m}B_{i}(Z)F_{i}(Z)\right)}^{r+1} \end{array}$$

where the PDF for the effective duration of the backoff period *B_i_*(*Z*) and sensing failure *F_i_*(*Z*) are derived as the following equations. The PGF of the time for backoff decrement process from first stage to *ith* stage can be expressed by the product of these (*i* + 1) stages. A node can choose a random backoff counter *W_i_* in stage *i* and each with probability 1/*W_i_* for uniform distribution of counter, that is, the node chooses counter as 0 with probability 1/*W_i_*, or 1 with probability 1/*W_i_* and so on, and PGF of each backoff process is the sum of all possibilities. Sensing failure time consists of two parts: CCA1 and CCA2, and the channel is busy with probabilities of *α* and (1 − *α*)*β*, respectively. CCA1 is performed after backoff counter decreased to zero, and CCA2 is only performed as channel being sensed idle after CCA1 with probability of (1 − *α*). Either of CCAs fails, and the node increases one backoff stage until the maximum stage:

$$B_{i}(Z)=\prod_{k=0}^{i}\sum_{l=0}^{W_{k}}\frac{1}{W_{k}}Z^{l}=\prod_{k=0}^{i}\frac{1}{W_{k}}\frac{1-Z^{W_{k}}}{1-Z},F_{i}(Z)=\sum_{k=0}^{i}\alpha^{k}Z^{k}\sum_{k=0}^{i}{\left((1-\alpha )\beta \right)}^{k}Z^{2k}$$

#### 5.2.2. Queuing Delay *T_q_*(*Z*)

**Figure 6.**
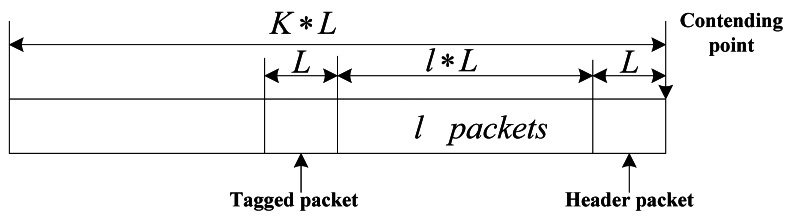
The relation between the tagged packet and the header packet in one tagged buffer. After a tagged packet arrives at the tagged queue, it has a distance *l* away from the header packet. It should wait for the time to be transmitted, which contains three parts.

We denote the discussed random arrival packet as a tagged packet in either type of nodes to account for packet queuing delay. Tagged packet has a distance of *l* packets away from the header packet of its queue when it arrives at the queue *N*_1_ if the analyses base on only *N*_1_, shown in Figure 6, denoted as *pak*_*N*_1__, and the queue which consists of tagged packet is called tagged queue accordingly. Queuing delay of *pak*_*N*_1__ consists of three parts: the time for transmitting (*l +* 1) packets in front of the tagged packet in this queue contained the header packet, the time for transmitting *K* packets in each of other (*N*_1_ − 1) nodes and the time for transmitting *K* packets in each of *N*_2_ nodes. Access delay for any non-tagged packet is the same as the tagged one analyzed as Equation (39).

According to the probability distribution of queue size at packet departure of Equations (26), (27) and the number of packet arrivals during packet access time, we derive expressions for tagged packet delay distribution at arbitrary time between departures related in [24,38,39]. General queue length distribution will be treated by a joint probability distribution of the number of packets in the device queue and the remaining service time for the packet which is currently being serviced. System steady state can be characterized by introduced three variables: *L_m_* which is denoted as the current queue length, *T_t−_* and *T_t+_* which is denoted as the elapsed service time and remaining service time for the current being serviced packet respectively. We can derive the joint probability distribution of queue size and remaining packet service time as follows:

$$\Gamma_{\text{ql}}^{*}(s)=\int_{0}^{\infty }e^{-sy}\mathrm{Pr}\text{ob}[L_{m}=l_{1,2},y<T_{t+}<y+dy]$$

Now, we consider the tagged queue length distribution based on only *N*_1_, and the same analysis process can be applied to that of *N*_2_. According to the PGF of accessing time, packets arriving at tagged queue *N*_1_ consist of two parts: the arrivals *l*_1_ if the current service packet belongs to (*N*_1_ − 1) and the arrivals *l*_2_ if the current service packet belongs to *N*_2_. The joint probability distribution consists of two parts as following Equations (40) and (41) [24,38]: arrival length *l*_1_ in mean packet access time $\bar{T_{tr1}}$ for type of *N*_1_ if the current service is one of *N*_1_ − 1 for the first part, and arrival length *l*_2_ in mean packet access time $\bar{T_{tr2}}$ for type of *N*_2_ if current service is one of *N*_2_:

(40) $$\begin{array}{l} \Gamma_{q(l_{1}+l_{2})}^{*}=\Gamma_{ql_{1}}^{*}+\Gamma_{ql_{2}}^{*}(1\leq l_{1,2}\leq K-1)\\ =\lambda_{1}\bar{T_{tr1}}(1-P_{K1})\mu_{01}E[e^{-sT_{1+}}e^{-\lambda_{1}T_{1-}}\frac{{\left(\lambda_{1}T_{1-}\right)}^{l_{1}-1}}{(l_{1}-1)!}]+\lambda_{1}\bar{T_{tr1}}(1-P_{K1})\sum_{j=1}^{l_{1}}\mu_{j1}E[e^{-sT_{1+}}e^{-\lambda_{1}T_{1-}}\frac{{\left(\lambda_{1}T_{1-}\right)}^{l_{1}-j}}{(l_{1}-j)!}]\\ +\lambda_{1}\bar{T_{tr2}}(1-P_{K1})\mu_{01}E[e^{-sT_{2+}}e^{-\lambda_{1}T_{2-}}\frac{{\left(\lambda_{1}T_{2-}\right)}^{l_{2}-1}}{(l_{2}-1)!}]+\lambda_{1}\bar{T_{tr2}}(1-P_{K1})\sum_{j=1}^{l_{2}}\mu_{j1}E[e^{-sT_{2+}}e^{-\lambda_{1}T_{2-}}\frac{{\left(\lambda_{1}T_{2-}\right)}^{l_{2}-j}}{(l_{2}-j)!}] \end{array}$$

(41) $$\begin{array}{l} \Gamma_{\text{qK}}^{*}(s)=\lambda_{1}\bar{T_{tr1}}(1-P_{K1})\mu_{01}\sum_{l_{1}=K-1}^{\infty }E[e^{-sT_{1+}}e^{-\lambda_{1}T_{1-}}\frac{{\left(\lambda_{1}T_{1-}\right)}^{l_{1}}}{l_{1}!}]+\lambda_{1}\bar{T_{tr1}}(1-P_{K1})\sum_{j=1}^{K-1}\mu_{j1}\sum_{l_{1}=K-j}^{\infty }E[e^{-sT_{1+}}e^{-\lambda_{1}T_{1-}}\frac{{\left(\lambda_{1}T_{1-}\right)}^{l_{1}}}{l_{1}!}]\\ +\lambda_{1}\bar{T_{tr2}}(1-P_{K1})\mu_{01}\sum_{l_{2}=K-1}^{\infty }E[e^{-sT_{2+}}e^{-\lambda_{1}T_{2-}}\frac{{\left(\lambda_{1}T_{2-}\right)}^{l_{2}}}{l_{2}!}]+\lambda_{1}\bar{T_{tr2}}(1-P_{K1})\sum_{j=1}^{K}\mu_{j1}\sum_{l=K-j}^{\infty }E[e^{-sT_{2+}}e^{-\lambda_{1}T_{2-}}\frac{{\left(\lambda_{1}T_{2-}\right)}^{l_{2}}}{l_{2}!}] \end{array}$$

The probability that *l*_1_ arrive at queue *N*_1_ during the service time of *N*_1_ and the probability that *l*_2_ arrive at queue *N*_1_ during the service time of *N*_2_ are derived as following Equation (42) [39], respectively:

(42) $$\begin{array}{c} \psi_{l_{1}}^{1*}(s)=E[e^{-sT_{1+}}e^{-\lambda_{1}T_{1-}}\frac{{\left(\lambda_{1}T_{1-}\right)}^{l_{1}}}{l_{1}!}]=\frac{\lambda_{1}}{\bar{T_{tr1}}}(T_{tr1}^{*}(s){\left(\frac{\lambda_{1}}{\lambda_{1}-s}\right)}^{l_{1}+1})-\sum_{k=0}^{l_{1}}a_{k1}{\left(\frac{\lambda_{1}}{\lambda_{1}-s}\right)}^{l_{1}+1-k})\\ \psi_{l_{2}}^{2*}(s)=E[e^{-sT_{2+}}e^{-\lambda_{1}T_{2-}}\frac{{\left(\lambda_{1}T_{2-}\right)}^{l_{2}}}{l_{2}!}]=\frac{\lambda_{1}}{\bar{T_{tr2}}}(T_{tr2}^{*}(s){\left(\frac{\lambda_{1}}{\lambda_{1}-s}\right)}^{l_{2}+1})-\sum_{k=0}^{l_{2}}a_{k1}{\left(\frac{\lambda_{1}}{\lambda_{1}-s}\right)}^{l_{2}+1-k}) \end{array}$$

Equations (40) and (41) can be simplified by substituting Equation (42) into them as following Equations (43) and (44) (0 ≤ *l*_1,2_ ≤ *K* − 1), in which the first factor denotes *l*_1_ arriving at queue *N*_1_ during the service time of *N*_1_ and the second factor denotes *l*_2_ arriving at queue *N*_1_ during the service time of *N*_2_:

(43) $$\Gamma_{q(l_{1}+l_{2})}^{*}(s)=\lambda_{1}\bar{T_{tr1}}(1-P_{K1})(\mu_{01}\psi_{l_{1}-1}^{1*}(s)+\sum_{j=1}^{l_{1}}\mu_{j1}\psi_{l_{1}-j}^{1*}(s))+\lambda_{1}\bar{T_{tr2}}(1-P_{K1})(\mu_{01}\psi_{l_{2}-1}^{2*}(s)+\sum_{j=1}^{l_{2}}\mu_{j1}\psi_{l_{2}-j}^{2*}(s))$$

(44) $$\Gamma_{\text{qK}}^{*}(s)=\lambda_{1}\bar{T_{tr1}}(1-P_{K1})(\mu_{01}\sum_{l=K-1}^{\infty }\psi_{l_{1}}^{1*}(s)+\sum_{j=1}^{K-1}\mu_{j1}\sum_{j=K-j}^{\infty }\psi_{l_{1}}^{1*}(s))+\lambda_{1}\bar{T_{tr2}}(1-P_{K1})(\mu_{01}\sum_{l=K-1}^{\infty }\psi_{l_{2}}^{2*}(s)+\sum_{j=1}^{K-1}\mu_{j1}\sum_{j=K-j}^{\infty }\psi_{l_{2}}^{2*}(s))$$

So, we can substitute Equation (42) and Equations (26), (27) into Equations (43), (44) to obtain the following expressions:

(45) $$\begin{array}{l} \Gamma_{q(l_{1}+l_{2})}^{*}(s)=(1-P_{K1})(T_{tr1}^{*}(s)(\mu_{01}{\left(\frac{\lambda_{1}}{\lambda_{1}-s}\right)}^{l_{1}}+\sum_{j=1}^{l_{1}}\mu_{j1}{\left(\frac{\lambda_{1}}{\lambda_{1}-s}\right)}^{l_{1}-j+1})-{\sum_{j=0}^{l_{1}-1}\mu_{j1}(\frac{\lambda_{1}}{\lambda_{1}-s})}^{l_{1}-j})\\ +(1-P_{K1})((T_{tr2}^{*}(s)(\mu_{01}{\left(\frac{\lambda_{1}}{\lambda_{1}-s}\right)}^{l_{2}}+\sum_{j=1}^{l_{2}}\mu_{j1}{\left(\frac{\lambda_{1}}{\lambda_{1}-s}\right)}^{l_{2}-j+1})-{\sum_{j=0}^{l_{2}-1}\mu_{j1}(\frac{\lambda_{1}}{\lambda_{1}-s})}^{l_{2}-j}) \end{array}$$

(46) $$\begin{array}{l} \Gamma_{\text{qK}}^{*}(s)=\frac{\lambda_{1}}{s}(1-P_{K1})(T_{tr1}^{*}(s)(\mu_{01}{\left(\frac{\lambda_{1}}{\lambda_{1}-s}\right)}^{K-1}+\sum_{j=1}^{K-1}\mu_{j1}{\left(\frac{\lambda_{1}}{\lambda_{1}-s}\right)}^{K-j})-{\sum_{j=0}^{K-1}\mu_{j1}(\frac{\lambda_{1}}{\lambda_{1}-s})}^{K-1-j})\\ +\frac{\lambda_{1}}{s}(1-P_{K1})(T_{tr2}^{*}(s)(\mu_{01}{\left(\frac{\lambda_{1}}{\lambda_{1}-s}\right)}^{K-1}+\sum_{j=1}^{K-1}\mu_{j1}{\left(\frac{\lambda_{1}}{\lambda_{1}-s}\right)}^{K-j})-{\sum_{j=0}^{K-1}\mu_{j1}(\frac{\lambda_{1}}{\lambda_{1}-s})}^{K-1-j}) \end{array}$$

So, we can derive the LST (Laplace-Stieltjes transform) of service delay. Service time is the time from packet arriving at nodes to departure, and we assume that the probability distribution of the queue length at packet arriving epoch is the same as the probability distribution of the queue length at arbitrary epoch [24], so service delay of two queues consists of three parts: the time for transmitting *l* packets and current being serviced header packet in front of the tagged packet, the time for transmitting *l*_1_(*l*_1_ ≤ *K*) packets in each of (*N*_1_ − 1) queues and the time for transmitting *l*_2_(*l*_2_ ≤ *K*) packets in each of *N*_2_ queues, which shown in Figure 6. Substituting Equations (26), (27) and Equation (30) into Equation (47), the packet service delay can be derived using a numerical method [24]:

(47) $$T_{q1}^{*}(s)=\frac{T_{tr1}^{*}(s)}{1-P_{K1}}(P_{0}+\sum_{l_{1}=1}^{K-1}\Gamma_{ql_{1}}^{*}(s){\left(T_{tr1}^{*}(s)\right)}^{l_{1}-1})+\frac{T_{tr2}^{*}(s)}{1-P_{K1}}(P_{0}+\sum_{l_{2}=1}^{K-1}\Gamma_{ql_{2}}^{*}(s){\left(T_{tr2}^{*}(s)\right)}^{l_{2}-1})$$

As we consider the tagged packet in *N*_2_, we can derive the service time distribution as that of tagged packet in *N*_1_ by substituting the queue parameters of *N*_2_ for that of *N*_1_ in a similar way. For example, joint probability distribution consists of two parts as shown in the following Equations (48), (49): *l*_1_ arrives at tagged queue *N*_2_ in mean packet access time $\bar{T_{tr1}}$ of *N*_1_ if the current service is one of *N*_1_ for the first part, and *l*_2_ arrives at tagged queue *N*_2_ in mean packet access time $\bar{T_{tr2}}$ of *N*_2_ if current service is one of *N*_2_ − 1. Other results can be derived as the similar way as that of *N*_1_, and we can omit this repetitive process for $T_{q2}^{*}(s)$:

(48) $$\begin{array}{l} \Gamma_{q(l_{1}+l_{2})}^{*}=\Gamma_{ql_{1}}^{*}+\Gamma_{ql_{2}}^{*}\\ =\lambda_{2}\bar{T_{tr1}}(1-P_{K2})\mu_{02}E[e^{-sT_{1+}}e^{-\lambda_{2}T_{1-}}\frac{{\left(\lambda_{2}T_{1-}\right)}^{l_{1}-1}}{(l_{1}-1)!}]+\lambda_{2}\bar{T_{tr1}}(1-P_{K2})\sum_{j=1}^{l_{1}}\mu_{j2}E[e^{-sT_{1+}}e^{-\lambda_{2}T_{1-}}\frac{{\left(\lambda_{2}T_{1-}\right)}^{l_{1}-j}}{(l_{1}-j)!}]\\ +\lambda_{2}\bar{T_{tr2}}(1-P_{K2})\mu_{02}E[e^{-sT_{2+}}e^{-\lambda_{2}T_{2-}}\frac{{\left(\lambda_{2}T_{2-}\right)}^{l_{2}-1}}{(l_{2}-1)!}]+\lambda_{2}\bar{T_{tr2}}(1-P_{K2})\sum_{j=1}^{l_{2}}\mu_{j2}E[e^{-sT_{2+}}e^{-\lambda_{2}T_{2-}}\frac{{\left(\lambda_{2}T_{2-}\right)}^{l_{2}-j}}{(l_{2}-j)!}] \end{array}$$

(49) $$\begin{array}{l} \Gamma_{\text{qK}}^{*}(s)=\lambda_{2}\bar{T_{tr1}}(1-P_{K2})\mu_{02}\sum_{l_{1}=K-1}^{\infty }E[e^{-sT_{1+}}e^{-\lambda_{2}T_{1-}}\frac{{\left(\lambda_{2}T_{1-}\right)}^{l_{1}}}{l_{1}!}]+\lambda_{2}\bar{T_{tr1}}(1-P_{K2})\sum_{j=1}^{K-1}\mu_{j2}\sum_{l_{1}=K-j}^{\infty }E[e^{-sT_{1+}}e^{-\lambda_{2}T_{1-}}\frac{{\left(\lambda_{2}T_{1-}\right)}^{l_{1}}}{l_{1}!}]\\ +\lambda_{2}\bar{T_{tr2}}(1-P_{K2})\mu_{02}\sum_{l_{2}=K-1}^{\infty }E[e^{-sT_{2+}}e^{-\lambda_{2}T_{2-}}\frac{{\left(\lambda_{2}T_{2-}\right)}^{l_{2}}}{l_{2}!}]+\lambda_{2}\bar{T_{tr2}}(1-P_{K2})\sum_{j=1}^{K}\mu_{j2}\sum_{l=K-j}^{\infty }E[e^{-sT_{2+}}e^{-\lambda_{2}T_{2-}}\frac{{\left(\lambda_{2}T_{2-}\right)}^{l_{2}}}{l_{2}!}] \end{array}$$

### 5.3. Energy Consumption Analysis

Energy consumption is the most important metric in WSNs, and we also analyze it elaborately. We assume a node is sleeping in a backoff period while it is receiving in extra waiting period after a successful transmission or not. Thus, we assume a node does not consume any energy during backoff procedures. Moreover, energy consumption of turnaround process *P_ta_* can be simplified to (*P_RX_* + *P_TX_*)/2, and energy consumption between two consecutive CCA attempts can also be simplified to this value. We can assume each packet transmission consumes the same energy, and mean energy consumptions for packet transmission are derived as Equation (50):

(50) $$\begin{array}{l} E_{\text{tot}}^{n}=(P_{\text{RX}}+P_{\text{ta}})\sum_{j=0}^{r}\sum_{i=0}^{m}\left(b_{i,0,j}^{n}+b_{i,-1,j}^{n}\right)+(P_{\text{TX}}+P_{\text{ta}})\sum_{j=0}^{r}\sum_{k=0}^{L-1}\left(b_{-1,k,j}^{n}+b_{-2,k,j}^{n}\right)+P_{\text{RX}}\sum_{j=0}^{r}\sum_{k=0}^{L_{\text{ex}}-1}(b_{-1,k,j}^{n}+b_{-2,k,j}^{n})+P_{\text{ta}}L_{\text{ta}}\sum_{j=0}^{r}\sum_{i=0}^{m}(1-\beta )b_{i,-2,j}^{n}\\ =(P_{\text{RX}}+P_{\text{ta}})\tau_{n}+(P_{\text{RX}}+P_{\text{ta}})(1-\alpha_{n})\tau_{n}+P_{\text{TX}}L\tau_{n}(1-\alpha_{n})(1-\beta_{n})+P_{\text{RX}}L_{\text{ex}}\tau_{n}(1-\alpha_{n})(1-\beta_{n})+P_{\text{ta}}L_{\text{ta}}\tau_{n}(1-\alpha_{n})(1-\beta_{n}) \end{array}$$

$$E_{\text{tot}}=N_{1}KE_{\text{tot}}^{1}+N_{2}KE_{\text{tot}}^{2}$$

## 6. Model Validations

Now we present extensive simulations of slotted IEEE 802.15.4 to validate our scheme with heterogeneous and unsaturated traffic using the NS-2 simulator [43]. NS-2 is a popular discrete-event simulator which was originally designed for wired networks and has been subsequently extended to support wireless simulations. The accuracy of evaluated expressions for throughput, delay and energy consumption is validated through extensive comprehensive simulations which are derived based on analyses of different parameters such as packet arrival rate, packet size and the node distributions.

Randomly deployed in a circle area of radius 3 meters with one sink in the center receiving data, nodes are all in the range of each other transmitting packets to sink. The transmission range of the transceiver is about 7 m. Node model is initiated as related in [41]. We assume that the entire superframe duration is active, moreover, the effect of beacon receptions set to one backoff period can be neglected for the beacon concluded in the data packet occupies a very small fraction time in a beacon order of 4. We assume each turnaround process consumes the same time and energy for simplification. Parameters used in simulations are listed in Table 1. Experimental setups of NS-2 simulator used to conduct validations are similar to presentations in [44] in detail, and the propagation delay can be ignored in our scheme simulations. We validate the performance of our analyses firstly, and then we compare the performance of our schemes with that of previous schemes such as Ramachandran's [12] and Sarmiento's [31]. Our simulation results are mean values derived from 20 experience values for each scenario.

Packet length is fixed to 7 units of backoff period including the PHY-header and MAC-header period. Backoff stage and retry number are fixed to 5 and 3, respectively. We study the asymmetry or heterogeneity and buffer characteristics of OSTS/BSTS schemes in this paper. We consider that the relative arrival rates *λ*_1_, *λ*_2_ and relative numbers *N*_1_, *N*_2_ which represent the system asymmetry and heterogeneity, and parameter *K* which represent the system capacity, play important roles on system metrics such as throughput, mean service delay and energy consumption. Furthermore, we can also derive system fairness from these metrics.

Performance is evaluated as the function of the aggregate offered load in different system size. Two different network sizes, *N* = 10 and *N* = 25, are considered, and the most heterogeneous distribution *N*_1_ = 13, *N*_2_ = 12 and the least one *N*_1_ = 23, *N*_2_ = 2 of the network of *N* = 25 are also considered under the same system offered load. Performance metrics are both sensitive to arrival rates *λ*_1_, *λ*_2_ and numbers *N*_1_, *N*_2_ for the same buffer capacity, and we cannot derive the variation tendency if these four parameters change at the same time without any datum mark. In this way, we can evaluate the performance as the functions of R = *λ*_1_/*λ*_2_ in the datum mark of system aggregate offered load, which is fixed at each buffer length of system size *N*. In particular, we can define normalized aggregate offered load as *G* = *g*_0_(*N*_1_*λ*_1_ + *N*_2_*λ*_2_), in which the parameter *g*_0_ is an impact factor standing for adjustment of system size and transmission arrival rate. Preferable and comprehensive performance metrics as the functions of ln*R* are evaluated through simulations. Differentiation of some set *λ*_1_, *λ*_2_ may be intuitively large, such as ln*R* = −2 means that *λ*_1_ = 0.135*λ*_2_, but performance differentiation can be evened largely through node distribution of *N*_1_ and *N*_2_ in the same offered load. Performance curves are manifested to smoothness character in function of ln *R* in our numerical analysis, which is also related in [25].

### 6.1. Throughput Validation

**Figure 7.**
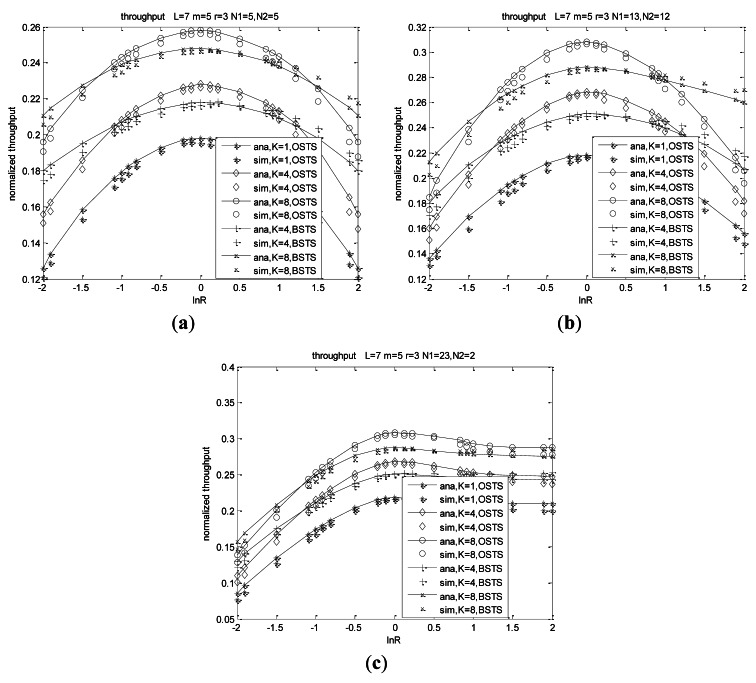
Throughput as a function of *λ*_1_/*λ*_2_ for fixed loads; (**a**) Normalized throughput of the most heterogeneous condition for a size of *N* = 10; (**b**) Normalized throughput for the most heterogeneous network of a size of *N* = 25; (**c**) Normalized throughput for the least heterogeneous network of a size of *N* = 25.

According to Equation (37), system throughput is determined by packet length, idle period and transmission probabilities in a normalized form in one CPC. This is because transmitted packets are always the header packets of the queues, in which accessing packets are the same as those of the no-buffer system. Figure 7 shows throughput characters as the functions of ln*R*. We can obtain throughput nature based on two aspects: asymmetry and heterogeneity.

We firstly consider the asymmetry characters for the same heterogeneity conditions shown in Figure 7(a,b). In the same transmission scheme, throughput increases with the asymmetry decreasing which means that throughput increases with the difference of arrival rates decreasing, and arrives at its peak value when *λ*_1_ is equal to *λ*_2_. Node's buffer capacity also plays an important role on throughput illustrated in Figure 7(a–c). With *K* increasing, throughput increases for the pending packets increasing. We consider that throughput and other metrics such as service delay and energy efficiency in the case of *K* = 1 for BSTS scheme are the same as those of the case of *K* = 1 for OSTS scheme, and we can also consider the packet length of BSTS scheme as *KL*. Throughput for OSTS shows different characteristics from those of BSTS for the different buffer capacity. Throughput of OSTS increases more rapidly with the difference of packet arrival rates decreasing at the same buffer size in that the probability of channel for BSTS scheme sensing busy increases with the difference of arrival rates decreasing. Nodes in BSTS spend much time to wait for being transmitted for the relative long service packets, which leads to transmission inefficient. Moreover, the retransmitted packets accumulate at the boundary of the superframe for offered load increases because FIMP algorithm presented in [40] is adopted in our BSTS scheme. With the number of nodes increasing, throughput also increases at the same ln *R* for the offered load increasing.

Heterogeneity also plays a decisive role on system performance shown from the curves of Figure 7(b,c). Throughput increases as the heterogeneity increases at ln*R* < 0, while throughput decreases with the heterogeneity increasing at ln*R* > 0, meaning that throughput of *N*_1_ = 13, *N*_2_ = 12 is higher than that of *N*_1_ = 23, *N*_2_ = 2 at each ln*R* when ln*R* < 0, while throughput of *N*_1_ = 13, *N*_2_ = 12 is lower than that of *N*_1_ = 23, *N*_2_ = 2 at each ln*R* when ln*R* > 0. Note that ln(*λ*_1_/*λ*_2_) = 0 means a network consisting of *N*_1_ + *N*_2_ identical nodes for *λ*_1_ equal to *λ*_2_ for a fixed network size, leading to each curve passing through the same point at *λ*_1_/*λ*_2_ = 1 for the same load, which can be seen from the point of ln(*λ*_1_/*λ*_2_) = 0 in Figure 7(b,c). For example, throughput is 0.268 for OSTS scheme and 0.251 for BSTS scheme at ln(*λ*_1_/*λ*_2_) = 0 when *K* = 4 in network *N*_1_ = 13, *N*_2_ = 12, which is the same value of *N*_1_ = 23, *N*_2_ = 2 for OSTS and BSTS, respectively, and throughput is 0.308 for OSTS scheme and 0.288 for BSTS scheme at ln(*λ*_1_/*λ*_2_) = 0 when *K* = 8 in both distribute networks, respectively. Moreover, throughput of *N*_1_ = 23, *N*_2_ = 2 in our system increases to a saturated value with *λ*_1_/*λ*_2_ increasing, and then decreases with a marginal rate shown in Figure 7(c). We observe that the predictions of these models are consistent with simulation results. For OSTS, we can observe that simulation values are lower than analysis results, and the deflection is of 3.542% to 6.334%. Those simulation values of BSTS are lower than analysis values when ln(*λ*_1_/*λ*_2_) < 0, while simulation results are higher than analysis results when ln(*λ*_1_/*λ*_2_) > 0, and the deflection is of 2.659% to 5.645%. These deflections are sustainable to our applications.

### 6.2. Delay Validation

Delay is the most important character in our real-time monitoring system, and we always attempt to improve the behavior of delay in order to obtain the real-time monitoring. From Equations (39) and (47) in Section 5.2, mean delay for transmitting a packet is mostly related to system asymmetry and heterogeneity, along with the buffer size for the same offered load and system scale. For a small size of network *N*_1_ + *N*_2_ = 10, mean delay is much lower than that of large size such as *N*_1_ + *N*_2_ = 25 for nodes increasing results in more pending packets and more collisions.

**Figure 8.**
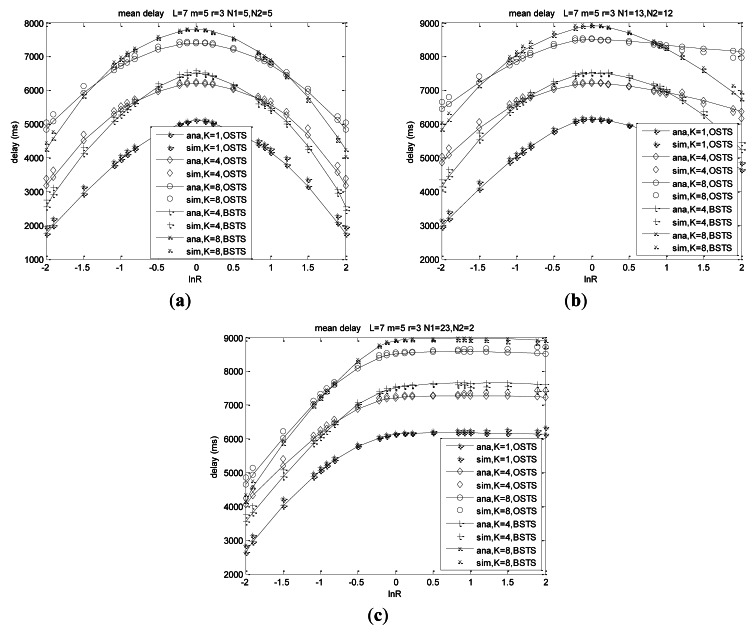
Delay as a function of *λ*_1_/*λ*_2_ for fixed loads. (**a**) Mean delay for a size of *N* = 10; (**b**) Mean delay for the most heterogeneous network of a size of *N* = 25; (**c**) Mean delay for the least heterogeneous network of a size of *N* = 25.

Asymmetry and heterogeneity play decisive roles on the system delay performance from the curves in Figure 8. Mean delay increases with the asymmetry decreasing which means the absolute value of ln*R* decreasing, and reaches its peak when traffic rate *λ*_1_ is equal to *λ*_2_ at different node distributions shown in Figure 8(a,b). Mean delay increases with buffer size *K* increasing for more offered load, more failure packets and consequently more retransmission. When the arrival rates differ much from each other, mean delay of BSTS scheme is lower than that of OSTS scheme at the same system size and same traffic intensity, and then it increases higher than that of OSTS scheme when the difference of packet arrival rates decreases. Adopting the scheme of FIMP for BSTS scheme, nodes wait for a long time to complete the transmission of *KL* packets which is a large length relative to WSNs, and then detect indicators of packet segments to find out retransmission packets, which consumes much time, leading to delay for BSTS higher than that of OSTS in this case.

Heterogeneity also plays a decisive role on delay performance shown from these curves. Delay increases with the heterogeneity of the network increasing at ln*R* < 0, while delay decreases with the heterogeneity increasing at ln*R* > 0 shown in Figure 8(b,c), meaning that delay of *N*_1_ = 13, *N*_2_ = 12 is higher than that of *N*_1_ = 23, *N*_2_ = 2 at each ln*R* when ln*R* < 0, while delay of *N*_1_ = 13, *N*_2_ = 12 is much lower than that of *N*_1_ = 23, *N*_2_ = 2 at each ln*R* when ln*R* > 0. Mean delay is less sensitive to ln*R* when nodes of *N*_1_ are much more than those of *N*_1_ (such as *N*_1_ = 23, *N*_2_ = 2) and traffic rate of *λ*_1_ is much more than that of *λ*_2_, which means system packets almost consisting with only *N*_1_ and delay performance is almost determined by traffic rate *λ*_1_ of *N*_1_, which is presented from the comparison of Figure 8(b,c). Mean delay arrives at the same values for the same load when ln(*λ*_1_/*λ*_2_) = 0 for the network composed of *N*_1_ + *N*_2_ identical nodes related as above.

**Figure 9.**
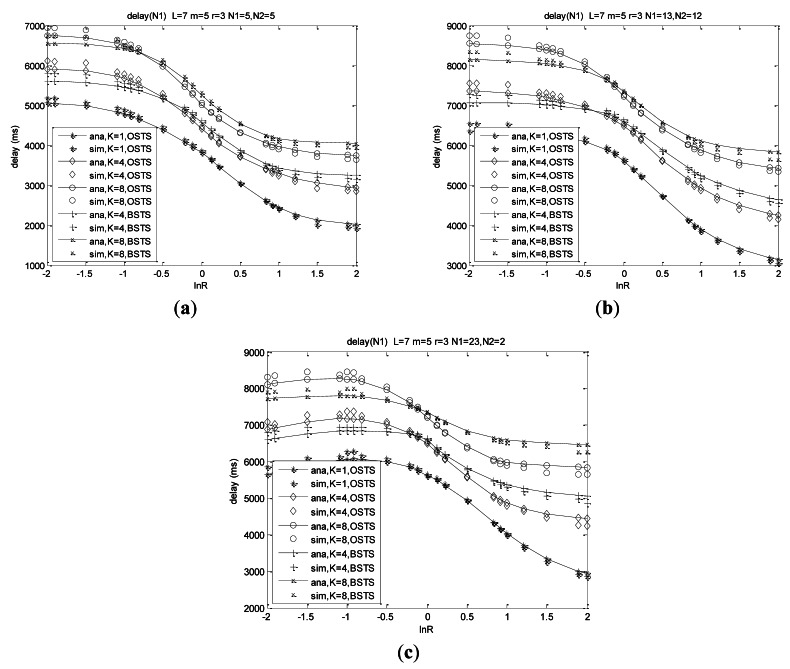
Delay for each system size of *N*_1_ as a function of *λ*_1_/*λ*_2_ for fixed loads. (**a**) Delay for a size of *N* = 10; (**b**) Delay for the most heterogeneous network of a size of *N* = 25; (**c**) Delay for the least heterogeneous network of a size of *N* = 25.

We can observe that respective delay of *N*_1_ and *N*_2_ are not similar to the characters of system total mean delay. Asymmetry and heterogeneity also play important roles on the respective delay behaviors observed from the curves in Figure 9. Delay of *N*_1_ decreases with increasing *λ*_1_/*λ*_2_, and its rate of decrease increases with the decreasing asymmetry. Delay of *N*_1_ at *N* = 10 for OSTS scheme is more than that of BSTS scheme when ln*R* < −0.495 in the case of *K* = 4, while delay of *N*_1_ for OSTS scheme is less than that of BSTS scheme when ln*R* > −0.495 shown in Figure 9(a,b).

In case of *K* = 8, delay of *N*_1_ for OSTS scheme is more than that of BSTS scheme when ln*R* < −0.865, while the delay of *N*_1_ for the OSTS scheme is less than that of BSTS scheme when ln*R* > −0.865. Delay of *N*_1_ increases with the increasing buffer capacity, and also increases with the network scale. Shown in Figure 9(b,c), the delay of *N*_1_ is insensitive to the heterogeneity for ln*R* < 0, while it decreases with the heterogeneity increasing for ln*R* > 0. The respective curves pass through the same point at ln(*λ*_1_/*λ*_2_) = 0 for the same load.

**Figure 10.**
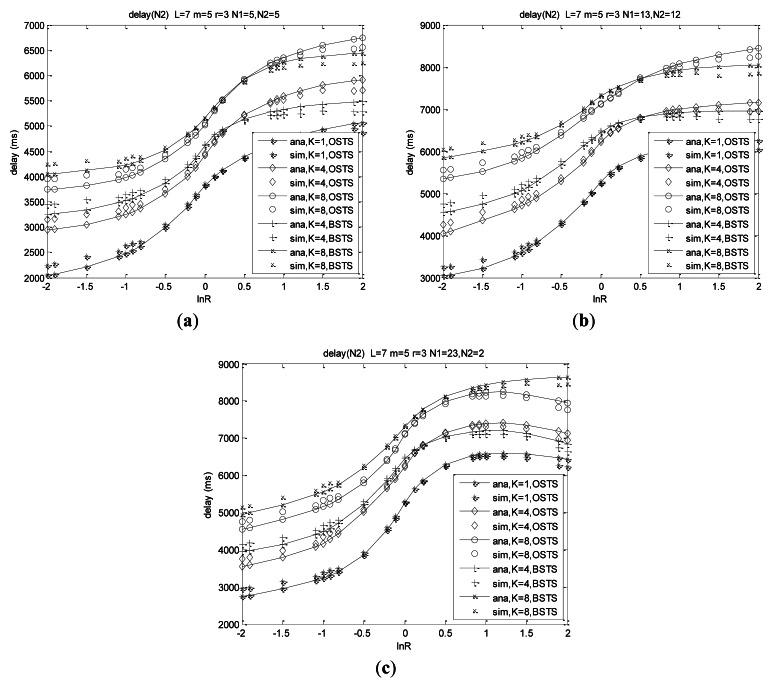
Delay for each system size of *N*_2_ as a function of *λ*_1_/*λ*_2_ for fixed loads. (**a**) Delay for a size of *N* = 10; (**b**) Delay for the most heterogeneous network of a size of *N* = 25; (**c**) Delay for the least heterogeneous network of a size of *N* = 25.

Delay analysis of *N*_2_ is similar to that of *N*_1_. We can observe that delay of *N*_2_ increases with increasing *λ*_1_/*λ*_2_, and its rate of increase increases with the decreasing asymmetry as shown in Figure 10. Delay of *N*_2_ at *N* = 10 for BSTS scheme is more than that of the OSTS scheme when ln*R* < 0.407 in the case of *K* = 4, while the delay of *N*_2_ for the BSTS scheme is less than that of the OSTS scheme when ln*R* > 0.407 as shown in Figure 10(a,b). In case of *K* = 8 at *N* = 10, the delay of *N*_2_ for the BSTS scheme is more than that of the OSTS scheme when ln*R* < 0.501, while the delay of *N*_2_ for the BSTS scheme is less than that of the OSTS scheme when ln*R* > 0.501. The delay of *N*_2_ for other node distributions can also be analyzed as shown in Figure 10(b,c). The delay of *N*_2_ increases with the increasing buffer capacity, and also increases with the network scale. Heterogeneity plays a similar role on delay of *N*_2_ as on delay of *N*_1_.

As shown in Figure 10(b,c), the delay of N_2_ is insensitive to the heterogeneity for lnR > 0, while it increases with the increasing heterogeneity for lnR < 0. The respective curves pass through the same point at ln(λ_1_/λ_2_) = 0 for the same load.

### 6.3. Energy Consumption Validation

**Figure 11.**
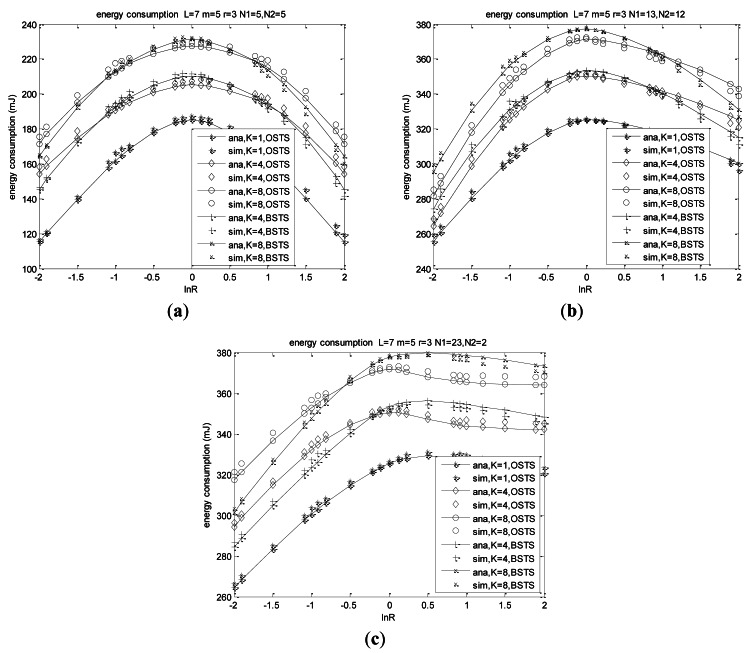
Energy consumption as a function of *λ*_1_/*λ*_2_ for fixed loads. (**a**) Energy consumption for a size of *N* = 10; (**b**) Energy consumption for the most heterogeneous network of a size of *N* = 25; (**c**) Energy consumption for the least heterogeneous network of a size of *N* = 25.

Energy is a most important factor considered in WSNs that withdraw energy from batteries, and it is also analyzed elaborately in our time-critical system. We assume that nodes are sleeping in the backoff procedure for energy efficiency, without any energy consumption. Energy analysis is similar to the throughput analysis in a small system size shown in Figure 11(a). Nodes in a system of *N* = 25 consume much energy than thaose of *N* = 10 regardless of the most heterogeneous *N*_1_ = 13, *N*_2_ = 12 or the least heterogeneous one, which is shown in Figure 11(b,c). Energy consumption is insensitive to the heterogeneity when ln*R* < 0, while sensitive to the node distribution when ln*R* > 0. In the case of the least heterogeneous network of *N*_1_ = 23, *N*_2_ = 2, much more energy is consumed for more packets generated by *N*_1_ with higher arrival rate *λ*_1_ when ln*R* > 0, and at the same time packets contributed by *N*_2_ are relatively high, which results in energy consumption always increasing shown in Figure 11(c).

**Figure 12.**
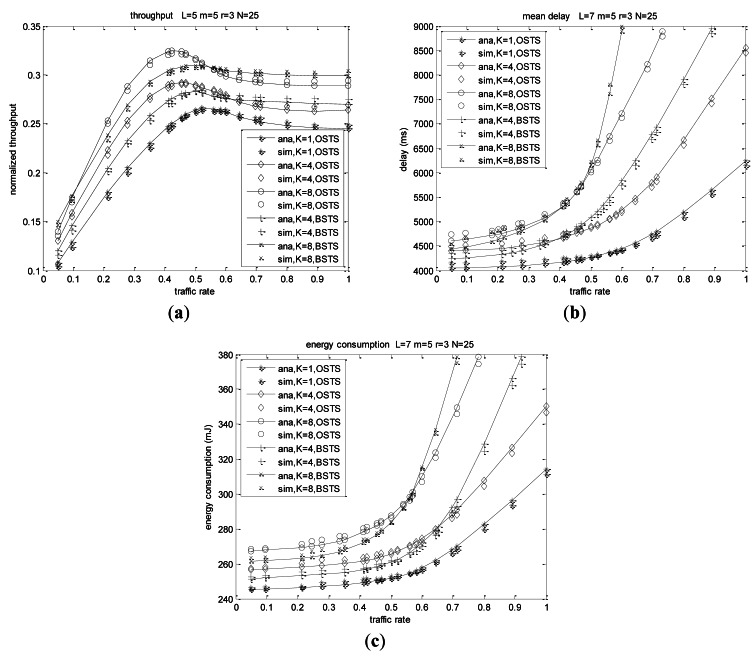
Performance when *λ*_1_/*λ*_2_. (**a**) Relations of throughput with buffer size and traffic rate; (**b**) Relations of mean delay with buffer size and traffic rate; (**c**) Relations of energy consumption with buffer size and traffic rate.

We also analyze system characteristics when the traffic rates are equal to each other, which means the homogeneous or symmetric condition mostly studied before [6,8,12]. Throughput increases rapidly for small values of the offered load, while it arrives at a peak point then decreases slightly or barely for large values of *G* as shown in Figure 12(a). The offered load will increase rapidly with increasing packet arrival rate, and successful transmission probability will increase slowly when more nodes are contending for the channel. Throughput of OSTS reaches a peak value foremost at *λ* = 0.573 for *K* = 1 and decreases slowly until a fixed value, while it reaches its peak at *λ* = 0.443 for *K* = 4 and at *λ* = 0.413 for *K* = 8, respectively. With packet arrival rate increasing, throughput of BSTS scheme reaches a peak value foremost at *λ* = 0.515 for *K* = 4 and decreases slowly to a fixed value, while it reaches its saturation at *λ* = 0419 for *K* = 8, respectively.

In the same way, mean delay will increase slowly for small offered load and rapidly for large *G* as shown in Figure 12(b). Due to less packets contending to transmit when the offered load is small, successful probability becomes high, so less energy consumption ensues. Pending packet accumulating at the beginning of superframe will lead to failed probability and collision probability increasing for offered load increasing, and energy consumption will increase rapidly for large *G*, as shown in Figure 12(c).

According to the analysis and simulation results, we observe that the heterogeneity and asymmetry play decisive roles in system behavior, and buffer size also impacts largely on the characteristics of the schemes. Performance metrics are demonstrated to have different superiority when adopting different transmission modes, OSTS or BSTS. We can choose the appropriate scheme of OSTS and BSTS according to node distribution of the applications as shown in Figures 7–12. The difference of packet arrival rates is very high, and the performance of BSTS is relatively superior to that of OSTS. Conversely, if the arrival rates are near to each other, the behavior of OSTS excels that of BSTS. We observe that the predictions of these models are consistent with simulation results. For OSTS, we can observe that simulation values of delay have a deflection of 5.634% to 8.242%, and simulation values of energy consumption have a deflection of 4.371% to 6. 427%. For BSTS, the deflections for delay and energy consumption are 4.843% to 7.475% and 4.845% to 7.968%, respectively. The deflections are suitable for our applications.

### 6.4. Performance Comparisons with Other Schemes

Analysis and simulation results shown above are comprehensive for applications, and we can compare the performance metrics of our mechanism with those of other non-priority heterogeneous schemes. Our schemes are used for time-critical monitoring and detection application, in which minimized delay is the most important target. Different types of nodes contend for the the channel with a fair chance, and the fairness is also an improved requirement. Adopting the distinguished improvement of taking the global viewpoint into account, our schemes OSTS/BSTS excel in WSN networks with non-priority traffic. Through the comprehensive comparisons, we can derive that the delay and fairness performance metrics of our schemes are obviously improved over other schemes such as [12,31], while throughput and energy efficiency are improved over others in more heterogeneous conditions.

A performance analysis model of the IEEE 802.15.4 CSMA/CA scheme with heterogeneous traffic is presented in [31], based on the viewpoint of the respective packet transmission of two types of nodes rather than based on the viewpoint of overall networks, which lead to the competitive packets of these two types being independent of each other. Correspondingly, our model analyzes the performance metrics based on the overall point of view, that is, the packets of two types of nodes are taken account for contending the channel with dependent interactions at the same time. This difference can be obviously expressed using Equation (8) in [31] and Equation (15) in this proposition. We can slightly adjust network parameters and MAC parameters for the model [31] in order to compare the performance metrics with those of our model, which is illustrated in later simulation figures.

The most representative model of CSMA/CA scheme based on IEEE 802.11 with non-priority heterogeneous traffic is presented in [28]. The post-backoff states are introduced to describe the unsaturated character, leading to the fixed idle states rather than dynamic arrival rate-dependent idle states. Moreover, post-backoff states also ensure there is at least one slot before a transmission, which is not similar to the IEEE 802.15.4 mechanism that there is at least one slot before a transmission. These differences lead to the performance comparisons between the IEEE 802.11 scheme of [28] with heterogeneous traffic and other heterogeneous IEEE 802.15.4 schemes such as OSTS/BSTS are not instructive. In comparison with another comprehensive IEEE 802.15.4 scheme presented in [12], Markov models are developed for the channel and node states respectively to determine the fractions of time that a node spends in different states which are then used to determine the throughput and energy consumption characteristics, which can be instructive to our scheme improvements. A geometric random distribution is used to present the number of backoff slots rather than the uniform random distribution as many schemes such as our work or the work of [2], and the initialization of CW with 1 is developed to improve throughput. The scheme assumptions and other application specifications in [12] are similar to those of our schemes, and we can modify this homogeneous IEEE 802.15.4 CSMA/CA scheme as a heterogeneity-like CSMA/CA scheme, denoted as Ramachandran's scheme simply. We evaluate the behaviors of this modified network which combines with another type of node, working as the BSTS scheme. We compare the behaviors of these two IEEE 802.15.4 schemes of [12,31] with our schemes under low traffic rates, and modify the backoff counters as no limitation when increasing of backoff stages for all four schemes. Data length is fixed to seven backoff periods, and the other simulation parameters are presented as the same as our above simulations. CW value of Ramachandran's scheme is denoted as 2 in our comparisons, and the energy models of OSTS/BSTS are developed more comprehensively compared to Ramachandran's seen from Section 5. For the same node distribution, the heterogeneity can be described as the asymmetry.

**Figure 13.**
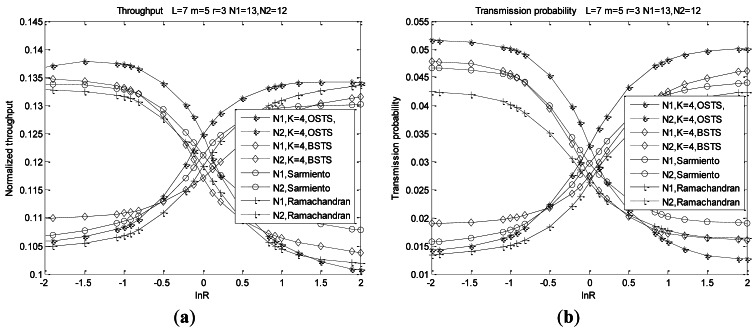
(**a**) Bandwidth share comparisons for four schemes using throughput metric. (**b**) Bandwidth share comparisons for four schemes using transmission probability metric.

We can present the access fairness comparisons based on the metrics of respective throughput and transmission probability according to the definition of [28], which considers the scheme to be fair if each node achieves a long-term throughput that is at least either its demand or a1/N share of the total achieved throughput. Throughput and transmission probability of *N*_1_ are the same as those of *N*_2_ when *λ*_1_ = *λ*_2_ shown in Figure 13(a,b), respectively, which demonstrates the fairness of the system. When the packet arrival rate of a type of node is much lower than that of the other, throughput of the lower one is much lower than that of the other, which denotes that the system is far from being fair. With the difference of packet arrival rate between these two types of nodes decreasing, the fairness of the system increases.

We can see from Figure 13(a) that bandwidth share of BSTS is superior to others when the difference of arrival rate is high and that of OSTS scheme is superior to others when the difference of arrival rate is small for the same network distributions. For example, throughput of BSTS scheme for *N*_1_ and *N*_2_ is 0.1098 and 0.1338 at ln*R* = −2, respectively, while its throughput for *N*_1_ and *N*_2_ is 0.1120 and 0.1343 at ln*R* = −1.5, respectively. Bandwidth share of BSTS scheme is 0.8992/1 at ln*R* = −2 and 0.9241/1 at ln*R* = −1.5, which is superior to those of Sarmiento's 0.8721/1 at ln*R* = −2 and 0.9013/1 at ln*R* = −1.5 and Ramachandran's 0.8693/1 at ln*R* = −2 and 0.8892/1 at ln*R* = −1.5 respectively. Bandwidth share of OSTS scheme is 1/1.0483 at ln*R* = 1, which is superior to those of Sarmiento's 1/1.0872 at ln*R* = 1 and Ramachandran's 1/1.1131 at ln*R* = 1. Packet length of Sarmiento's scheme *L_Sar_* or Ramachandran's scheme *L_Ram_* can be considered as *KL*, which is similar to that of the BSTS scheme. Shown from Figure 13(a), the bandwidth share of Sarmiento's is somewhat superior to that of OSTS when the difference of arrival rates is high. Less cooperation among packets of two types shown in Equation (8) of [31] brings out higher transmission efficiency for pending packets are almost dominated by the higher rate nodes for high asymmetry, and fair characters are derived for node transmits all its packets smoothly once it obtains the channel. Bandwidth share of Sarmiento's is some superior to that of BSTS when the difference of arrival rates decreases. For high traffic load, node of Sarmiento's scheme gives up the whole current packet for unsuccessful transmission rather than transmitting failed fractions repeatedly which leads to transmission inefficient. Bandwidth share of Ramachandran's is somewhat inferior to those of BSTS and Sarmiento's when the difference of arrival rates is high for the scheme of Ramachandran's is essentially designed as a homogeneous scheme. We simply combine with another type of nodes in order to compare the performance with these schemes, which results in less behavior interaction with each other. The scheme fairness is also expressed by transmission probability which is presented in Figure 13(b).

**Figure 14.**
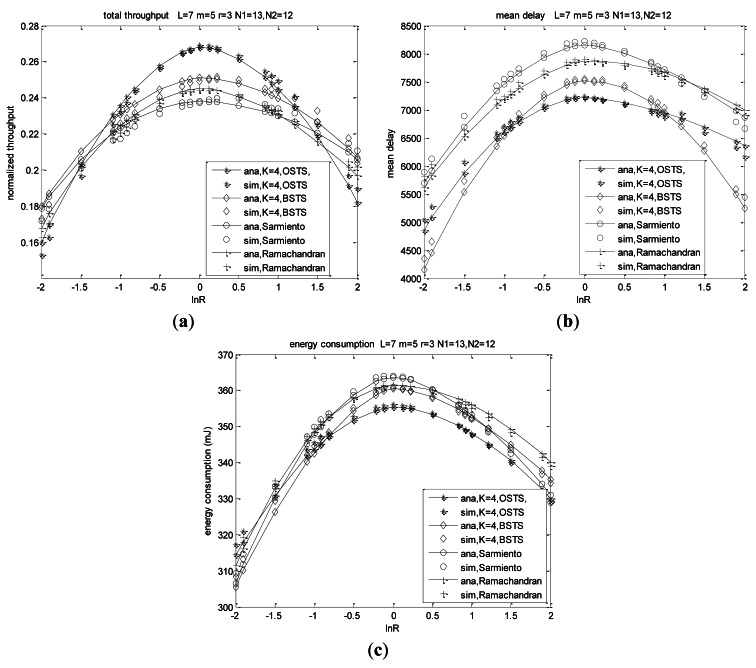
(**a**) Throughput comparisons for different schemes; (**b**) Mean delay comparisons for different schemes; (**c**) Energy consumption comparisons for different schemes.

Throughput comparisons are shown in Figure 14(a), and we find that throughput of Sarmiento's scheme is some superior to that of OSTS and BSTS when the heterogeneity decreases, such as in the case of |ln*R*| > 1.446 for OSTS and |ln*R*| > 2 for BSTS. When pending packets are dominated by one type of node with higher arrival rates, higher transmission efficiency is derived by Sarmiento's model for less cooperation between two types of nodes. When the heterogeneity increases, weaken cooperative transmission of Sarmiento's model brings out more collisions, and then throughput decreasing. Throughput of Ramachandran's scheme is always lower than that of BSTS, and is some superior to that of OSTS when the heterogeneity decreases to the case of ln*R* > 1.525 and ln*R* < −1.506 shown in Figure 14(a).

We find that delay metrics of OSTS and BSTS scheme are superior to those of Sarmiento's and Ramachandran's schemes from Figure 4(b) in three aspects. Normalized probability of Markov chain model is presented based on the single type of nodes rather than two types of nodes in Sarmiento's scheme, which leads to operating point parameters overestimation. Nodes transmit packets adopting independent transmission probability rather than the interacted one, which leads to the respective transmission probability underestimation. Moreover, idle process is simply presented by a fixed length rather than a queuing distribution and packet transmission is described without adopting the queuing theory, and we adopt the packet length *L_Sar_* or *L_Ram_* is simple expressed as *KL* which is a very large value for the IEEE 802.15.4 transmission scheme. Such a long packet transmission or retransmission consumes much waiting time for other pending packets, which leads to transmission inefficient and then delay increases. With the difference of arrival rate increasing which denotes traffic rate is relatively low, nodes in Ramachandran's scheme need to wait for a longer time to transmit the next frame since they shut down the radio for energy efficiency, resulting in more time consumption compared to the schemes OSTS/BSTS. With the difference of arrival rate decreasing, the time consumed for the radio switches decreases in Ramachandran's scheme, which results in the delay difference between Ramachandran's scheme and that of OSTS/BSTS decreasing.

Ramachandran's scheme consumes less energy than that of OSTS in the case of ln*R* < −1.672 for the total traffic rate is relatively low, which results in energy efficiency for the former scheme illustrated in Figure 14(c). With the difference of the traffic rate decreasing denoted traffic rate as relatively high, energy efficiency of Ramachandran's scheme decreases in that the receiver shuts down its radio frequently. Analysis results match well with simulation results seen from Figures 13 and 14.

We can find that the heterogeneous performance is improved greatly by adopting the novel schemes of OSTS/BSTS, in which the contending traffic, regardless of type, has no priority over each other. Delay and fairness of OSTS/BSTS are superior to those of other schemes, while throughput and energy efficiency are superior to others in more heterogeneous situations. In such a fair-required time-critical system, the schemes of OSTS/BSTS supply a satisfactory performance.

## 7. Conclusions

In this paper, two transmission schemes—OSTS and BSTS—are proposed to improve the performance of heterogeneous unsaturated networks. At first, accurate and comprehensive analyses for these two slotted CSMA/CA scheme using two semi-Markov and one macro-Markov models are made, along with a queuing model. These models contain a finite number of terminals and ideal channel, in which each node has a finite buffer capacity of *K* packets and each packet contains *L* units' backoff period. The probability of the buffer being empty at a departure is not the same with that of a random period, which has not been analyzed before under the heterogonous condition. Throughput, packet delay and energy consumption of heterogeneous and unsaturated networks are predicted, in which packets have non-preemptive priority over each other. Validity of the analytical model demonstrates that its predictions closely match the simulation results, and the heterogeneity and asymmetry play decisive roles on the performance. Homogeneous performance is also analyzed if the network transfers to symmetric condition. Moreover, performance comparisons between OSTS/BSTS schemes with other heterogeneous schemes are presented under fair transmission conditions. Analysis and simulation results demonstrate that our schemes improve the performance of service delay and contending fairness obviously, meanwhile, throughput and energy efficiency are improved largely at the most heterogeneous conditions.

Moreover, we should gain deep insights into several problems in our future works. We know that MAC sublayer needs a finite amount of time to process data received by the PHY. To allow for this, two successive frames transmitted from a device shall be separated by at least an InterFrame Spaces (IFS) period. If the first transmission requires an ACK, the separation between the ACK frame and the second transmission shall be at least an IFS period. Two frames are seldom transmitted successively from a device in these schemes and no ACK is contained in them, therefore, the IFS between two frames can be ignored in our OSTS/BSTS schemes. The IFS should be taken into account for the appropriate successive transmissions/receptions or ACK transmissions in the future studies. Then, it is worth noting that the CFP is considered as the solution to delay-sensitive applications such as video services, and this time-critical mean can be used for our real-time applications in the future. Also, the distances among nodes are relatively close, and the propagation signal effect can be omitted in our current representations. However, the nodes which are used to sense the quantities to be measured can be away from each other for larger distances, and several situations should be taken into considerations. Firstly, the propagation model should be included into the simulation results, which is a major deviation of the analysis and simulation results. Secondly, the distances among nodes go to such an extent as to transmit the packet in two or more hops, which brings out hidden terminals or more complicated pending problems. Furthermore, heterogeneous queues should be resolved by the relay nodes and the coordinator, respectively. Currently, our research focuses on such intractable multi-hop access problems, and we shall devote ourselves to study and then improve the behaviors of these multi-hop wireless sensor networks with buffered heterogeneous traffic in our forthcoming research.

## Figures and Tables

**Table 1.** The parameters of our simulations.

*aNumSuperframeSlots*	*960 symbols*	*L_data_* (Packet length)	2,240 μs
*aUnitBackoffPeriod*	*20 symbols*	*T_CCA_* (Time for CCA)	640 μs
*aBaseSlotDuration*	*60 symbols*	*T_slot_* (Slot duration)	320 μs
*DataRate*	*250 kbps*	*T_ta_* (Turnaround time)	12 symbols
*P_TX_*	17.4 mA	*P_RX_*	19.7 mA
*P_CCAs_*_(inte_*_rval_*_)_	18.5 mA	*P_ta_*	18.5 mA
